# SUMO-2 and PIAS1 Modulate Insoluble Mutant Huntingtin Protein Accumulation

**DOI:** 10.1016/j.celrep.2013.06.034

**Published:** 2013-07-18

**Authors:** Jacqueline Gire O’Rourke, Jaclyn R. Gareau, Joseph Ochaba, Wan Song, Tamás Raskó, David Reverter, John Lee, Alex Mas Monteys, Judit Pallos, Lisa Mee, Malini Vashishtha, Barbara L. Apostol, Thomas Peter Nicholson, Katalin Illes, Ya-Zhen Zhu, Mary Dasso, Gillian P. Bates, Marian Difiglia, Beverly Davidson, Erich E. Wanker, J. Lawrence Marsh, Christopher D. Lima, Joan S. Steffan, Leslie M. Thompson

**Affiliations:** 1 Department of Biological Chemistry, University of California, Irvine, Irvine, CA 92697, USA; 2 Department of Psychiatry and Human Behavior, University of California, Irvine, Irvine, CA 92697, USA; 3 Institute for Memory Impairments and Neurological Disorders, University of California, Irvine, Irvine, CA 92697, USA; 4 Structural Biology Program, Sloan-Kettering Institute, New York, NY 10065, USA; 5 Department of Neurobiology and Behavior, University of California, Irvine, Irvine, CA 92697, USA; 6 Department of Developmental and Cell Biology, University of California, Irvine, Irvine, CA 92697, USA; 7 Max-Delbrueck-Center for Molecular Medicine, 13125 Berlin, Germany; 8 Departments of Internal Medicine, Neurology, and Molecular Physiology and Biophysics, Roy J. and Lucille A. Carver College of Medicine, University of Iowa, Iowa City, IA 52242, USA; 9 Enzo Life Sciences (UK) Ltd., Palatine House, Matford Court, Exeter EX2 8NL, UK; 10 Laboratory of Gene Regulation and Development, National Institute of Child Health and Development, National Institutes of Health, Bethesda, MD 20892, USA; 11 Department of Medical and Molecular Genetics, King’s College London School of Medicine, London WC2R 2LS, UK; 12 Department of Neurology, Massachusetts General Hospital and Harvard Medical School, Charlestown, MA 02129, USA

## Abstract

A key feature in Huntington disease (HD) is the accumulation of mutant Huntingtin (HTT) protein, which may be regulated by posttranslational modifications. Here, we define the primary sites of SUMO modification in the amino-terminal domain of HTT, show modification downstream of this domain, and demonstrate that HTT is modified by the stress-inducible SUMO-2. A systematic study of E3 SUMO ligases demonstrates that PIAS1 is an E3 SUMO ligase for both HTT SUMO-1 and SUMO-2 modification and that reduction of *dPIAS* in a mutant *HTT Drosophila* model is protective. SUMO-2 modification regulates accumulation of insoluble HTT in HeLa cells in a manner that mimics proteasome inhibition and can be modulated by overexpression and acute knockdown of PIAS1. Finally, the accumulation of SUMO-2-modified proteins in the insoluble fraction of HD postmortem striata implicates SUMO-2 modification in the age-related pathogenic accumulation of mutant HTT and other cellular proteins that occurs during HD progression.

## INTRODUCTION

Huntington disease (HD) is caused by the expansion of a CAG repeat within the HD gene and the corresponding poly-glutamine track within the Huntingtin (HTT) protein (MacDonald et al., 1993). Symptoms include movement abnormalities, psychiatric symptoms, and cognitive deficits with accompanying degeneration of medium spiny neurons in striatum and loss of cortical volume (Ross and Tabrizi, 2011). Posttranslational modifications modulate protein function, and HTT is subject to multiple functionally relevant modifications including SUMOylation, ubiquitination, acetylation, palmitoylation, and phosphorylation (for review, see Ehrnhoefer et al., 2011; Pennuto et al., 2009). We previously demonstrated that a fragment of mutant HTT is modified by Small Ubiquitin-like MOdifier 1 (SUMO-1), and genetic reduction of SUMO in *Drosophila*-expressing mutant HTT exon 1 is protective (Steffan et al., 2004). SUMO-1 modification of mutant HTT in cells was also associated with increased toxicity and decreased aggregation by the striatal enriched small guanine nucleotide-binding protein Rhes (Subramaniam et al., 2009). SUMO modification is also implicated in Alzheimer’s disease (AD), Parkinson’s disease (PD), and amyotrophic lateral sclerosis (ALS), as well as other CAG repeat diseases (SBMA, DRPLA, SCA1, and SCA7) (for review, see Krumova and Weishaupt, 2013; La Spada and Taylor, 2010; Wilkinson et al., 2010). Although this modification is linked to pathogenesis, the precise mechanisms involved have not yet been elucidated.

**Figure 1:**
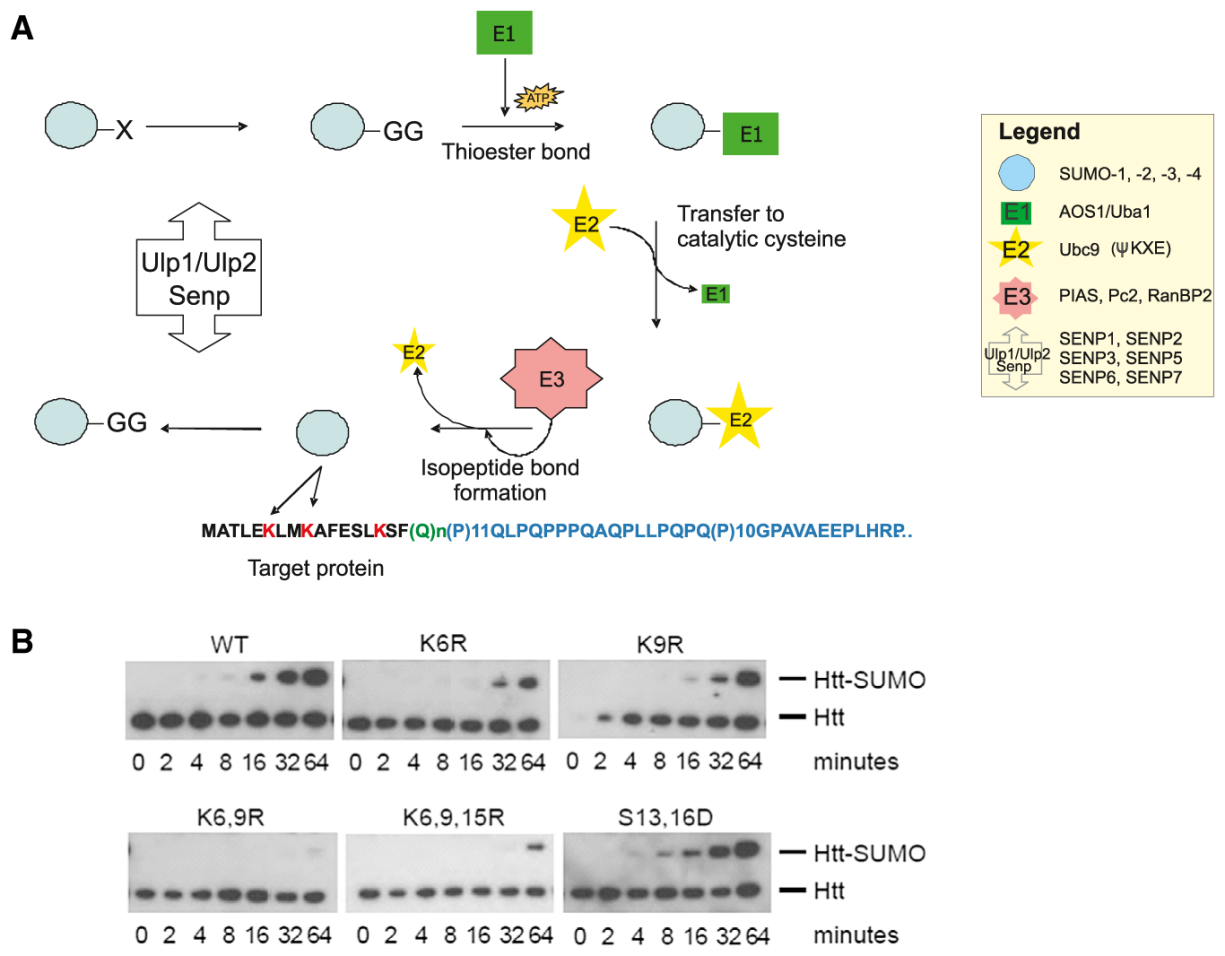
K6 and K9 Are Primary Sites for HTTex1p In Vitro SUMOylation (A) Schematic illustration of the SUMOylation pathway. SUMO is expressed as a precursor protein (SUMO-X) that is processed by SUMO-specific proteases (SENPs) to expose a C-terminal diglycine motif (-GG). Enzymatic reactions are similar to ubiquitination and include activation by the SUMO E1-activating enzyme (SAE1/UBA2), to the SUMO E2-conjugating enzyme (Ubc9), and transfer of SUMO to target lysines with or without the assistance of the SUMO E3-ligating enzymes. (B) Time course of SUMO-1 modification of His-tagged HTTex1p (25Q)-purified proteins (WT, K6R, K9R, K6,9R, K6,9,15R, and S13,16D) was performed in vitro. SUMOylation was visualized using anti-His antibody. WT HTTex1p is SUMOylated within 16 min, K6R or K9R mutations delay SUMOylation, and combined mutations (K6,9R or K6,9,15R) greatly reduce SUMOylation. Mutations that mimic phosphorylation (S13,16D) alter kinetics of SUMO-1 modification with SUMO modification observed beginning by 4 min. See also Figure S1.

SUMO modification is the covalent attachment of SUMO to specific lysine residues within a target protein and regulates key processes involved in normal cellular function, including subcellular localization, protein stability, transcriptional regulation, and interaction properties of SUMO-modified proteins with their cellular targets (Cubeñas-Potts and Matunis, 2013; Gareau and Lima, 2010). Although highly transient, the effects of this modification are long lasting (Johnson, 2004). Four different forms, SUMO-1–SUMO-4, exist in mammals. SUMO-2 and SUMO-3 are nearly identical (97% identity) and often referred to as one protein (SUMO-2/SUMO-3) (Gareau and Lima, 2010; Johnson, 2004), and SUMO-4 is found only in a precursor form (Bohren et al., 2004). The SUMOylation pathway involves a cascade of enzymes, similar to ubiquitination, with a single E1-activating enzyme (SAE1/UBA2), a single E2-conjugating enzyme (UBC9), multiple E3-ligating enzymes (Protein Inhibitors of Activated STAT [PIAS], PC2, MMS21, and RanBP2), which provide substrate specificity, and multiple pro-teases (SENPs) that both cleave the SUMO moiety from target proteins and process SUMO itself (Figure 1A). Given that SUMOylation is implicated in HD and other neurodegenerative diseases, identification of the E3s responsible for HTT modification may provide insight into mechanisms underlying HD and provide novel therapeutic targets.

Here, we systematically evaluated the enzymatic machinery involved in HTT SUMO modification and report for the first time that HTT is modified by SUMO-2 and that PIAS1 functions as a HTT SUMO E3 ligase. This modification may serve more than one function because longer HTT polypeptides, both wild-type (WT) and mutant, are SUMO modified downstream of exon 1. SUMO-2 overexpression causes mutant HTT to accumulate in cells. In HD postmortem striatum, SUMO-2-modified proteins accumulate in the insoluble fraction, suggesting that this modification is relevant in vivo to HD. Further validating the potential in vivo relevance for SUMO modification pathways in HD, genetic reduction of *dPIAS* in *Drosophila* expressing expanded repeat HTT is neuroprotective. Taken together, these results provide a rationale for targeting SUMO-2 and PIAS1 as novel therapeutic targets for HD.

## RESULTS

### HTTex1p Lys 6 and Lys 9 Are the Primary Sites of SUMO Modification

We previously showed that truncated HTT (Httex1p) is SUMO-1 modified in cells and that Lys 6 (K6) and Lys 9 (K9) may represent primary sites for modification based on the absence of SUMO modification upon mutagenesis of target lysines (Steffan et al., 2004). From these studies, it was not clear which lysines are preferentially SUMO modified. Based on SUMO prediction software (SUMOplot, Abgent), the lysines in HTTex1p (Figure 1A) do not fall within a classic SUMO consensus sequence but, rather, are low-probability SUMO sites or not predicted (Figure 5A). However, classic SUMO consensus sequences are neither necessary nor sufficient for determining SUMO modification of a protein. To directly determine which of the three N-terminal lysines (K6, K9, or K15) are preferentially SUMO modified, HTTex1p and lysine mutants, mutated singly and in combination, were purified and analyzed using an in vitro SUMO modification system followed by mass spectrometry analysis. SUMO-1 (t95R) was used based on ease of detection of mono-SUMO modification, and to minimize the confounding effect of aggregation, unexpanded HTTex1p (25Q) constructs were used. When K6 and K9 were mutated singly to arginine (K6R, K9R), SUMO modification is reduced compared to WT, but when K6 and K9 (K6,9R) are mutated together, SUMO modification is greatly reduced, similar to the three lysine mutants (Figure 1B), suggesting that K6 and K9 are the major target sites. Mass spectrometry analysis confirmed that K6 and K9 are indeed the primary sites of SUMO modification (Figure S1).

HTTex1p is subject to other posttranslational modifications such as ubiquitination and phosphorylation (Ehrnhoefer et al., 2011; Zheng and Diamond, 2012). Because (1) phosphorylation modulates SUMO modification of cellular proteins, (2) mimicking phosphorylation of serines 13 and 16 (S13 and S16, respectively) in expanded repeat HTTex1p (97QP) regulates SUMO-1 modification in cells (Thompson et al., 2009), and (3) this modification is relevant in vivo (Gu et al., 2009), we evaluated SUMO modification of WT HTTex1p (25Q) in the context of a HTT phosphomimic S13,16D in vitro. SUMO modification of the phosphomimetic HTT (25Q) was equal to or more rapid than for WT HTT control (Figure 1B); mass spectrometry analysis revealed that SUMO modification of the S13,16D phosphomimic is restricted to K6 (Figure S1). These results suggest that both in vitro and in cells, other posttranslational modifications, including phosphorylation, may influence SUMO modification of HTT.

### PIAS and SUMO Modification Proteins Are Highly Expressed in Mouse Brain

Because SUMO E3 ligases provide specificity in targeting proteins where a modified lysine does not fall within a consensus site (Gareau and Lima, 2010), such as HTT, identifying the E3 ligase(s) that promotes HTT SUMOylation may be key to identifying therapeutic targets that regulate this modification. Based on the fact that PIASy was identified as a HTT-interacting protein in a yeast two-hybrid (Y2H) screen (Goehler et al., 2004), we evaluated whether PIAS proteins could function as HTT E3 ligases. In humans, the PIAS family consists of four members: PIAS1, PIASx (xα and xβ), PIAS3, and PIASy. Originally identified as PIAS (Shuai and Liu, 2005), the PIAS proteins are involved in regulation of transcription, immune responses, cytokine signaling, and E3 ligase activity (Liu and Shuai, 2008; Rytinki et al., 2009). As E3 ligases, they enhance SUMOylation of a number of different proteins, and multiple PIAS proteins can sometimes act as E3 ligases for the same substrate (Schmidt and Müller, 2002).

**Figure 2:**
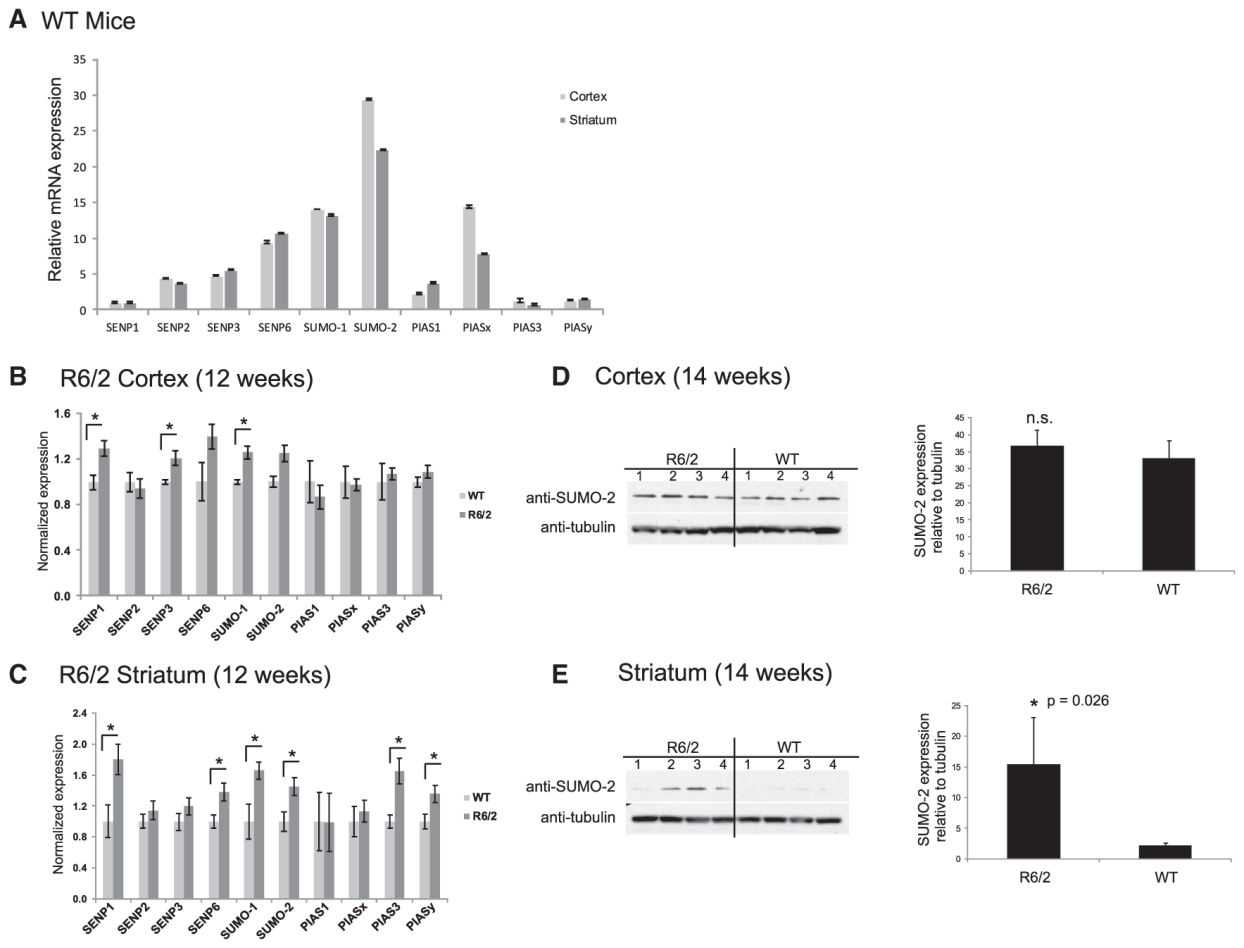
SUMO-1 and SUMO-2 Are Differentially Expressed in HD Mice versus Control (A) qRT-PCR analysis of SUMO-modifying proteins and enzymes in cortex and striata of WT mice at 12 weeks. Relative expression for all the SUMO enzymes is normalized to mouse β-actin. (B and C) qRT-PCR of SUMO mRNAs from 12-week-old WT and R6/2 mouse cortex (B) and striatum (C). SUMO enzyme mRNAs are differentially expressed in R6/2 versus control with statistically significant increases in *SENP1* (p = 0.01), *SENP3* (p = 0.02), and *SUMO-1* (p = 0.003) in cortex and *SENP1* (p = 0.02), *SENP6* (p = 0.04), *PIAS3* (p = 0.007), *SUMO-1* (p = 0.02), and *SUMO-2* (p = 0.02) in striatum. Samples were analyzed in quadruplicate and normalized to mouse β-actin. Data are shown as R6/2 expression relative to WT levels set at 1 for each enzyme with ± SD (n = 4). *p < 0.05. n.s., not significant. (D and E) Western blot analysis of SUMO-2 in 14-week R6/2 cortex (D) and striatum (E) versus aged-matched controls. SUMO-2 is upregulated in R6/2 striatum versus control (p = 0.026; n = 4). Protein is normalized to α-tubulin and quantitated using ImageJ. Note that only the 12-week time point is shown in (B) and (C). Please see 4- and 8-week time points in Figures S2A and S2B.

To first establish that SUMO modification enzymes are present in brain regions relevant to HD, expression of SUMO-related proteins was quantified in mouse striatum and cortex using quantitative RT-PCR (qRT-PCR). *SUMO-1*, *SUMO-2*, *PIAS*, and *SENP* mRNAs are all expressed in WT cortex and striatum. Within each region, *PIASx* is most highly expressed, followed by *PIAS1*, with *PIAS3* and *PIASy* having similar expression profiles (Figure 2A). This suggests that any PIAS could potentially serve as a HTT E3 SUMO ligase based on its expression in vivo. The SENP proteins are also expressed in the brain, with *SENP6* most highly expressed followed by *SENP2* and *SENP3*, and finally *SENP1* (Figure 2A). *SUMO-1* and *SUMO-2* are expressed in mouse brain at relatively high levels. These data demonstrate that the SUMO machinery is present in relevant brain regions.

To determine if these SUMO-related genes show expanded repeat HTT-dependent alterations in expression patterns, each was quantified in WT and R6/2 mouse cortex and striatum at 4, 8, and 12 weeks. R6/2 mice express a truncated HTT fragment (exon 1 with ~150Qs) and show very rapid HD-like disease progression with onset by approximately 6 weeks, highly penetrant phenotypes at 8 weeks, and end-stage disease by 12 weeks (Mangiarini et al., 1996). At 4 and 8 weeks, some dysregulation begins to occur (Figures S2A and S2B), and by 12 weeks, there are statistically significant increases of *SENP1*, *SENP3*, and *SUMO-1* in R6/2 cortex and of *SENP1*, *SENP6*, *PIAS3*, *SUMO-1*, and *SUMO-2* in R6/2 striatum (Figures 2B and 2C), suggesting that in vivo SUMO-modifying pathways may be perturbed in HD. The increases in *SUMO-1* and *SUMO-2* specifically in striatum, the region of greatest vulnerability to neurodegeneration, were particularly noteworthy. A similar pattern of increased SUMO-2 in R6/2 striatum but not cortex was also observed at the protein level (Figures 2D and 2E), suggesting a SUMO-2-selective response in striatum. RNA was also isolated from dissected brain regions of BACHD mice (Gray et al., 2008) that express full-length human HTT as a BAC transgene with 97Qs and show progressive disease over a longer time course. *SUMO-1* and *SUMO-2* expression was similarly increased in BACHD striatal samples at 14 months (Figure S2C), a time when disease phenotypes are evident, and *SUMO-2* is increased in N171 mouse striatum at 12 weeks compared to 6 weeks (Jia et al., 2012). The progressive nature of these changes, with the most robust observed at late disease stages, is consistent with the concept of early changes in protein homeostasis systems (Schipper-Krom et al., 2012) with later profound disruption of the SUMO network at the gene expression and protein level. Taken together, the most consistent change in fragment and full-length mutant HTT mouse models is upregulation of SUMO, suggesting that this pathway may be dysregulated in vivo.

### HTTex1p Is Modified by Both SUMO-1 and SUMO-2

**Figure 3:**
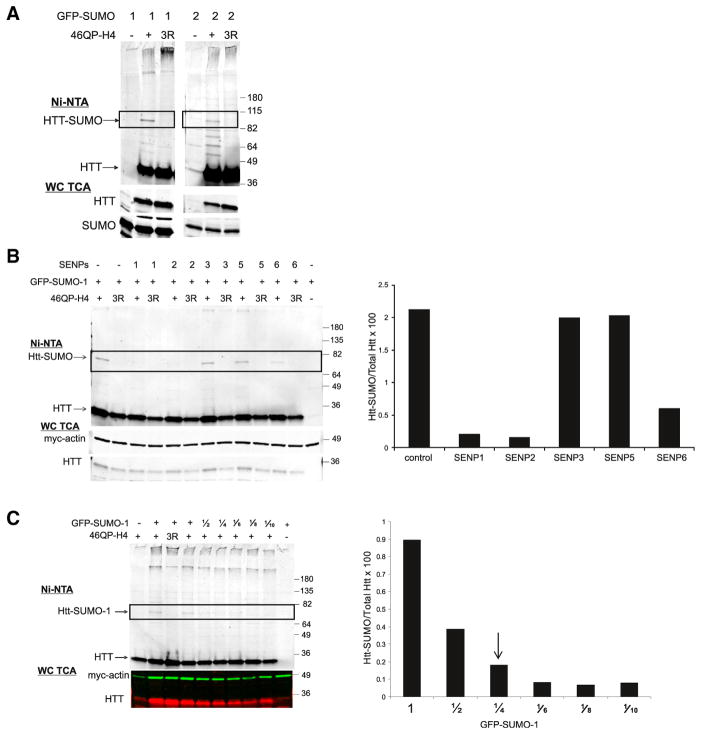
HTT Is Modified by Both SUMO-1 and SUMO-2 (A) HeLa cells transfected with His-tagged HTTex1p (46Q) or 46QP-K6,9,15R (3R) along with GFP-SUMO-1 or GFP-SUMO-2, lysed under denaturing conditions, and nickel purified (Ni-NTA). Unmodified HTT-46Q is indicated by the arrow and SUMO-modified HTT by the boxed region. The lysine mutant (3R) serves as a negative control. Ni-NTA represents nickel-purified His-tagged HTT, and WC TCA represents 10% of the whole-cell lysate expression of HTT and myc-actin (transfection control). HTT is modified by SUMO-1 (left) and SUMO-2 (right). (B) SUMO isopeptidases (SENP1, SENP2, SENP3, SENP5, and SENP6) modulate SUMO-modified HTT when overexpressed together with HTT (46QP-H4 or 3R) and SUMO-1 (GFP-SUMO-1). SENP1, SENP2, and SENP6 decrease HTT SUMOylation. Graph depicts quantitation of western blot using the Odyssey Infrared Imaging System (LI-COR) to calculate the ratio of HTT purified versus the HTT modified by SUMO multiplied by 100. (C) Titration of SUMO-1. Denaturing nickel purification of HTTex1p (46QP-H4) following transfection with decreasing amounts of SUMO-1 reduces the amount of SUMO-modified HTT to undetectable levels. The Ni-NTA blot displayed in the gray scale shows purified HTTex1 and SUMO-modified HTTex1 using HTT antibody. Note that 0.5 μg of SUMO-1 (¼ the amount of SUMO-1 cDNA ) was used for identifying the SUMO-1 E3 ligase for HTTex1p. Graph depicts quantitation of western blot using the Odyssey Infrared Imaging System to calculate the ratio of HTT purified versus the HTT modified by SUMO multiplied by 100. Note that all experiments were performed in triplicate, and a representative figure is shown. See also Figure S3A.

Modification by SUMO-2 is unique in its capacity to be induced by cellular stress (Saitoh and Hinchey, 2000). Based on our previous data that the stress-inducible kinase IKK can activate phosphorylation of HTT S13 and S16 and increase polySUMOylation of 97QP HTTex1p (Thompson et al., 2009), and given that oxidative stress and other cellular stressors are implicated in HD (Browne and Beal, 2006), we investigated whether SUMO-2 can modify HTT. A cell-based SUMOylation assay was optimized to visualize and quantify SUMO modification (Figure S3A; Extended Experimental Procedures), which for endogenous proteins, is a highly dynamic process, and only low levels of modification are typically observed for an individual protein at any given time (Johnson, 2004). Expanded repeat HTTex1p (46QP) with a C-terminal epitope tag (46QP-H4) was cotransfected with SUMO-1 (GFP-SUMO-1) or SUMO-2 (GFP-SUMO-2) and then purified under denaturing conditions using magnetic nickel beads (Ni-NTA). SUMO-1 and SUMO-2 can both modify HTTex1p, and when the lysines (K6, K9, and K15) are mutated to arginine (3R), SUMO modification cannot be detected (Figure 3A). An unusual laddering below control HTT in the presence of SUMO-2, but not in the presence of lysine mutants, is observed for this construct; however, the laddering is not detected by anti-GFP, and its significance is not clear.

### PIAS1 Is a Candidate SUMO E3 Ligase for HTT

Each of the PIAS proteins was evaluated for its ability to enhance SUMO modification of HTT, and the ratio of SUMO-modified HTT versus purified HTT was quantified using a western blot imaging system (Odyssey Imager; LI-COR). Because the PIAS proteins are regulators of transcription, like SUMO, and can act as coactivators and corepressors in addition to their E3 ligase activity (Rytinki et al., 2009), one-tenth mycactin under a CMV-based promoter was cotransfected to account for transcriptional effects (Thompson et al., 2009; Figure S3A). To control for differences in protein loading due to denaturing conditions precluding protein assays, each membrane was stained with a reversible protein stain (MEMCode). Finally, to control for differential HTT expression, 10% of each sample was trichloracetic acid (TCA) precipitated and subjected to western analysis. To demonstrate that the modified form of HTT does indeed represent SUMO-modified HTT, SUMO-1 was coexpressed in its mature, processed form (SUMO-1-GG) together with each of the SUMO isopeptidase SENPs (SENP1, SENP2, SENP3, SENP5, and SENP6) to show elimination by isopeptidases. SENP1, SENP2, and SENP6 each catalyzed removal of SUMO-1 from HTT, whereas SENP3 and SENP5 had no effect (Figure 3B), providing validation that the shift in protein mobility represents SUMO-modified HTT and suggesting selectivity of isopeptidase action.

**Figure 4:**
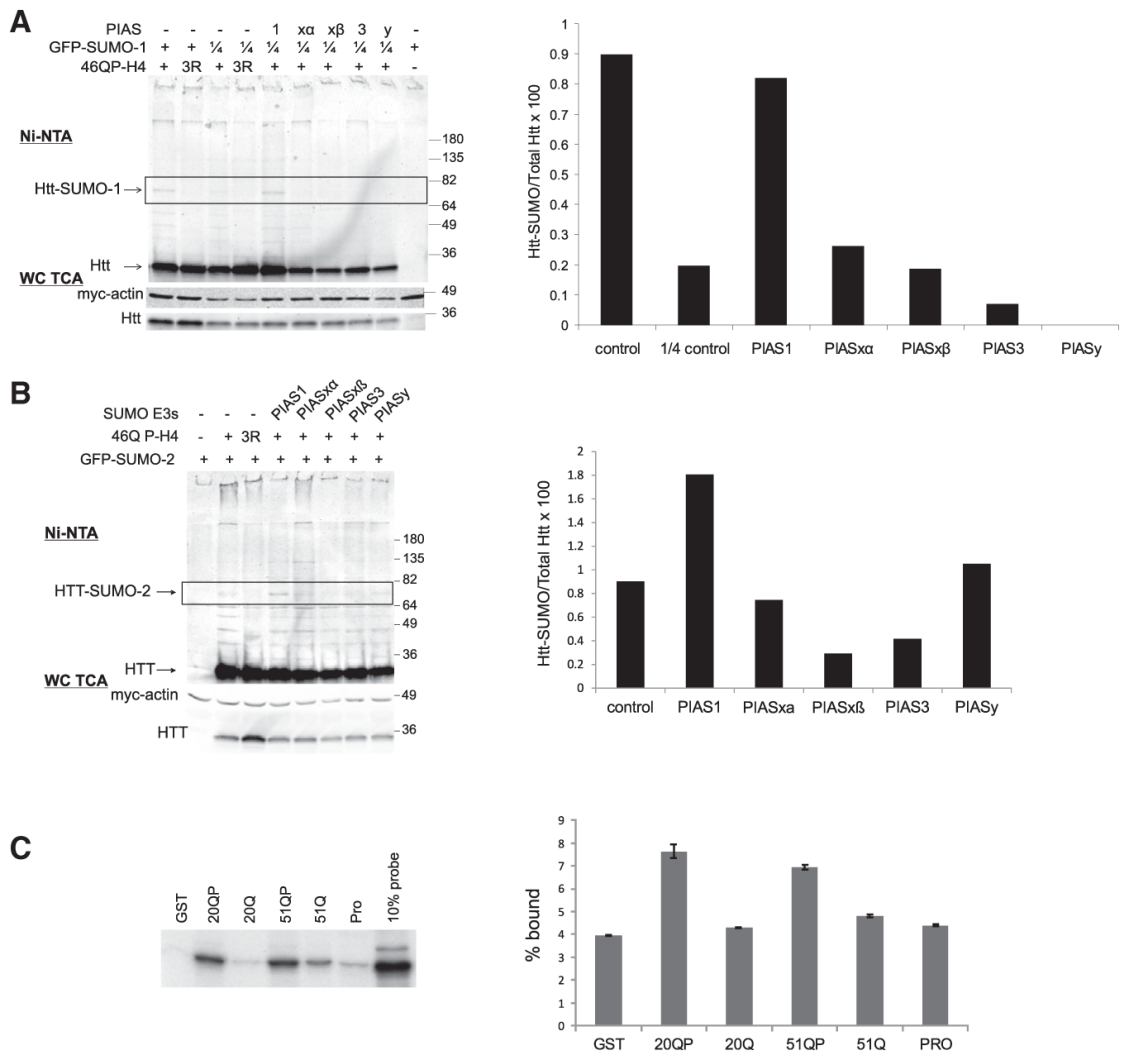
PIAS1 Is a SUMO E3 Ligase for HTT (A) Under limiting SUMO conditions (1/4 SUMO-1, lanes 3–9), PIAS1 increases HTT-SUMO modification above 1/4 SUMO alone. Purified HTT (arrow) and SUMO-HTT (boxed region) were detected using anti-HTT. Graph depicts quantitation of the Ni-NTA western blot using the Odyssey Infrared Imaging Software (LI-COR) to calculate the ratio of HTT purified versus the HTT modified by SUMO multiplied by 100. (B) Western analysis of overexpression of HTTex1 (46QP-H4 or 3R), SUMO-2 (GFP-SUMO-2), and all the PIAS proteins (PIAS1, PIASxα, PIASxβ, PIAS3, and PIASy). Under nonlimiting SUMO-2 conditions, PIAS1 enhances SUMO modification of HTT. WC TCA shows overall myc-actin (transfection control) and HTT levels. Graph quantitating the ratio of HTT purified versus the HTT modified using the Odyssey (LICOR). (C) Left panel is the autoradiography results of a GST pull-down assay showing that radiolabeled human PIAS1 interacts with HTTex1p. Right panel is a phosphorimager analysis of GST pull-downs, performed in triplicate, showing the percentage of 35S-labeled PIAS protein that bound the GST proteins: GST alone, unexpanded HTT with and without the proline-rich region (20QP and 20Q), expanded HTT with and without the proline-rich region (51QP and 51Q), and the proline-rich region alone (Pro). Error bars were calculated as an estimate of SE = STDEV(n)/SQRT(n-1).

To evaluate each PIAS protein for the ability to enhance HTT SUMO modification, 46QP-H4 was coexpressed with SUMO-1 (GFP-SUMO-1) and each of the PIAS proteins (PIAS1, PIASxα, PIASxβ, PIAS3, and PIASy). HTT is readily modified by SUMO-1 (Steffan et al., 2004) and (Figure 3A) at saturating levels of SUMO-1. In order to detect enhancement of SUMO-1 modification, a titration was performed to determine the SUMO-1 concentration at which modification of HTT was barely detectable (Figure 3C) and the addition of a relevant E3 ligase could increase HTT SUMO modification (Stankovic-Valentin et al., 2007). PIAS1 repeatedly enhanced SUMO-1 modification of HTTex1p (Figure 4A, representative figure), as did Rhes as previously reported (Figure S3B).

We next investigated whether SUMO-2 modification of HTT is also sensitive to the addition of an E3 ligase. Because this modification is more difficult to visualize under basal conditions, limiting SUMO-2 levels was not necessary. SUMO-2 was cotransfected with individual PIAS cDNAs, and PIAS1 was also most effective at enhancing SUMO-2 modification of HTT (Figure 4B, representative figure). In this assay, Rhes did not enhance the formation of a SUMO2-HTT species (Figure S3C).

Consistent with its role as an E3 SUMO ligase and the underlying rationale that direct interactions between E3 ligases and targets can promote SUMO modification, PIAS1 was evaluated for its ability to bind to HTT fragments in vitro. Using GST pull-down assays with HTT exon 1-encoding polypeptides, both normal range (20QP) and expanded repeat HTTex1p(51QP) interact strongly with PIAS1 (7.75% and 7%, respectively) (Figure 4C). As controls, protein lacking the protein-interacting proline-rich domain of Htt (20Q and 51Q) or expressing the proline-rich region alone showed greatly reduced interactions. These results suggest that a direct interaction between PIAS1 and HTTex1p may facilitate SUMO modification.

### Longer HTT Fragments Are SUMOylated

**Figure 5:**
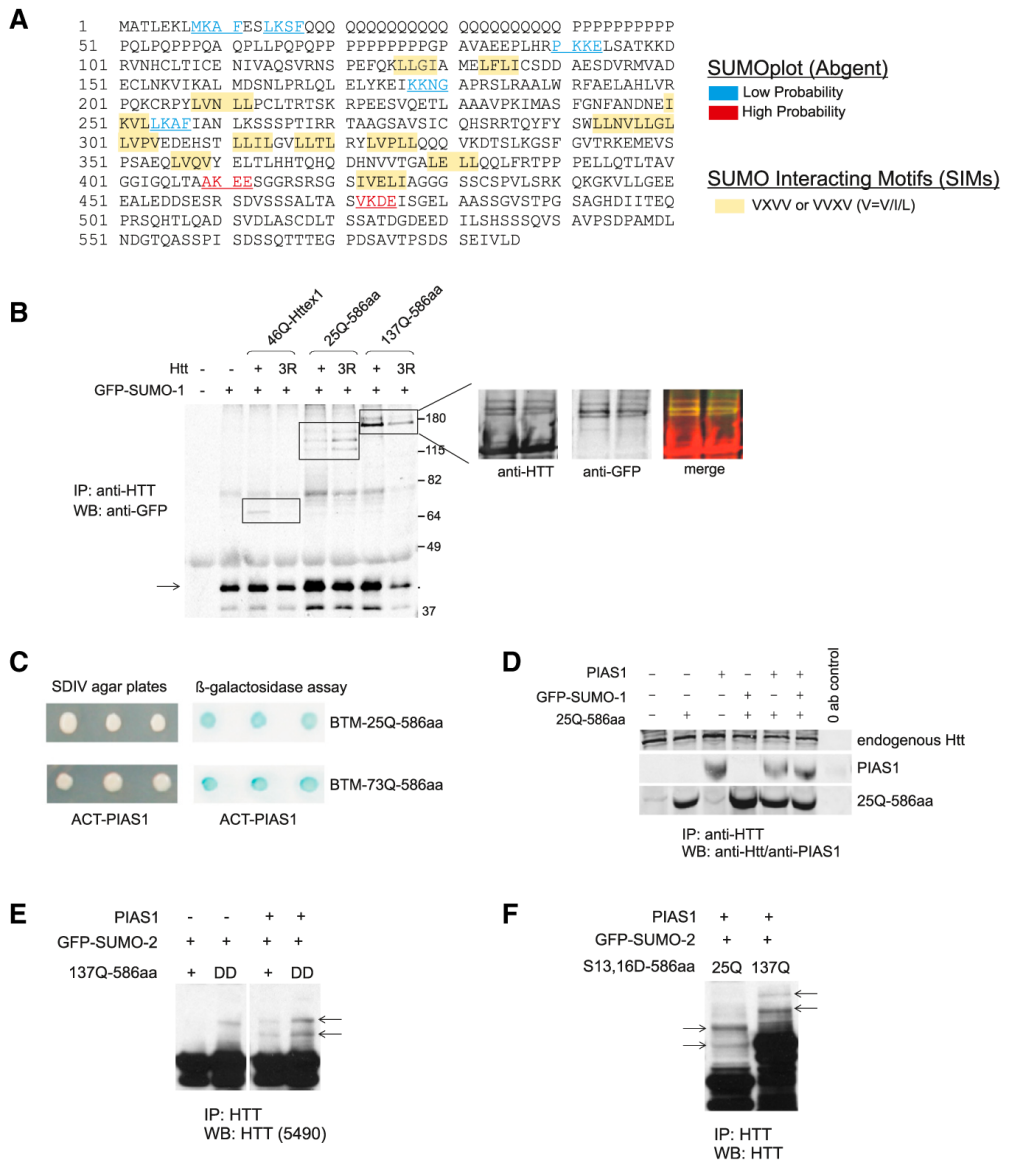
A 586 aa Fragment of HTT Is SUMO Modified Downstream of Exon 1 (A) SUMOplot (Abgent) predicts that HTT is SUMOylated downstream of HTTex1p lysines. SUMOplot analysis predicts two high-probability SUMO sites (red) and three low-probability SUMO sites (blue) identified downstream of HTT exon 1. Greater than 13 overlapping noncovalent SIMs are observed within this fragment (yellow). (B) Longer HTT polypeptides are modified by SUMO-1. Western blot overexpressing HTTex1p (46QP-H4 or 3R), unexpanded HTT-586 fragment (25Q-586 or 25Q-3R-586 aa), and expanded HTT-586 fragment (137Q-586 or 137Q-3R-586 aa) with SUMO-1 (GFP-SUMO-1). Cell lysates were subjected to HTT immunoprecipitation (IP) using HTT antibody. Western analysis performed with the Odyssey (LI-COR) allows detection of HTT (data not shown) and SUMO simultaneously and shows that all forms of HTT are SUMO-1 modified using the anti-GFP antibody. HTTex1p is covalently SUMO-1 modified (lane 3), and the modification disappears when Lys are mutated to Arg (3R) (lane 4). Both unexpanded (lane 4 and 5) and expanded HTT-586 fragments (lane 6 and 7) are covalently SUMO-1 modified in both the presence and absence of the three Lys in the N-terminal region of HTT (3R). Free SUMO-1 is indicated with the arrow, and SUMO-modified HTT is indicated by the boxes. Inset on the right, from a replicate experiment, shows comigration of expanded HTT (anti-HTT) and SUMO-1 (anti-GFP) displayed in the gray scale and in color when the two antibodies are merged (HTT in red, SUMO-1 in green, and yellow when colocalizing). (C) Bait plasmids (HTT-586-25Q or HTT-586-73Q aa) were transformed into the L40ccua MATa yeast strain. Yeast clones encoding bait proteins were individually mated against a matrix of MATα yeast clones encoding 16,888 prey proteins (with Gal4 activation domain fusions) using pipetting and spotting robots. Diploid yeasts were spotted onto SDIV (-Leu-Trp-Ura-His) agar plates for selection of PPIs as well as nylon membranes placed on SDIV agar plates for β-galactosidase assays. After 5–6 days of incubation at 30ºC, digitized images of the agar plates and nylon membranes were assessed for growth and β-galactosidase activity using the software Visual Grid (GPC Biotech). (D) Overexpression of PIAS1 alone, with unexpanded HTT constructs (25Q-586 aa) plus SUMO-1 (GFP-SUMO-1), shows that PIAS1 binds both full-length HTT and HTT-586 fragment (25Q-586 aa). WT HTT was used in these experiments to preclude confounding aggregation effects. Western analysis detection was performed using Odyssey and is displayed in the gray scale but is shown in color on the merge (HTT is red, and PIAS1 is green). WB, western blot. (E) HeLa cells overexpressing either expanded 586 aa-HTT or the phosphomimetic (S13,16D = DD) with SUMO-2 plus and minus PIAS1. HTT was purified by IP using hydrazide beads (Bioclone) crosslinked to HTT (Enzo) antibody and subjected to western analysis using anti-HTT (MAB5490). Arrows indicate SUMO-2-modified HTT. (F) IP of HTT with hydrazide-linked beads shows that both unexpanded and expanded HTT 586 aa phosphomimetics (S13, 16D-586 aa) are modified by SUMO-2. Arrows indicate SUMO-conjugated HTT. Note that all experiments including the Y2H assay were done in triplicate; representative experiments are shown. Arrows indicate SUMO-2-modified HTT.

A potentially critical and initiating cleavage event occurs at a caspase-6 cleavage site of HTT (Graham et al., 2006), creating a polypeptide of 586 amino acids (HTT 586 aa). Using bio-informatic tools (SUMOplot; Abgent), five additional lysines downstream of HTTex1p are predicted to be SUMO modification sites of high (two lysines) or lower (three lysines) probability (Figure 5A). Of interest, further analysis of the 586 aa fragment reveals up to 13 potential overlapping SUMO-interacting motifs (SIMs) within this region depending upon consensus sequence designation (Figure 5A) (Tatham et al., 2008). SIMs are noncovalent interactions that may enhance SUMOylation of the SIM-containing proteins themselves (Blomster et al., 2010). Therefore, the SUMO and SIMs in HTT may work together to regulate HTT SUMOylation.

To determine if longer HTT fragments are SUMOylated independently of the lysines within the first 17 aa, HTT 586 aa containing either an unexpanded (25Q-586 aa) or expanded polyQ repeat (137Q-586 aa) was coexpressed with SUMO-1 (Figure 5B) because this modification should be detected under basal conditions. As a control, HTT 586 aa constructs with K6,9,15R mutations that diminish SUMO modification within the first 17 aa were tested. Immunoprecipitated HTTex1p (46QP-H4) is monoSUMOylated by SUMO-1 (Figure 5B), but the 3R mutant form is not modified as expected. However, other lysines downstream of this amino-terminal domain can also be SUMOylated because SUMO-1 modification is observed even in the presence of the K6,9,15R mutation in either its expanded or unexpanded forms.

### PIAS1 Increases SUMO-2 Modification of HTT 586 aa

SUMO-2 modification of HTT 586 aa is not detected in the absence of external stimuli (Figure 5E); therefore, longer HTT fragments were evaluated for SUMO-2 modification in the presence of an E3-SUMO ligase. Potential interactions between longer HTT fragments and E3 SUMO ligases were first investigated using a large-scale Y2H screen. Here, the 586 aa fragment of HTT was used as bait, and PIAS1 emerged as the single E3 SUMO ligase interaction partner (Figure 5C), supporting its relevance even for longer HTT polypeptides. Previously identified interactors were also tested in this system and confirmed, including HIP2 and GIT1 (Goehler et al., 2004). To further validate this interaction in vitro, coimmunoprecipitation of HTT from cell lysates transfected with HTT (25Q-586) and PIAS1 with or without cotransfected SUMO-1 was tested. PIAS1 binds both transfected HTT (586 aa) and full-length endogenous HTT based on PIAS1 detection even in the absence of exogenous HTT 586 overexpression (Figure 5D). SUMO-2 modification of longer HTT polypeptides was therefore tested in the presence of PIAS1. Based on in vitro results (Figure 1B) and because SUMO-2 is stress inducible similar to the signal transduction cascades that modulate HTT phosphorylation, S13, 16D phosphomimic polypeptides were also tested. Expanded repeat HTT 586 aa fragments are SUMO-2 modified in the presence of PIAS1, and this modification is enhanced by mimicking phosphorylation (S13, 16D-586 aa) for both expanded and unexpanded HTT 586 aa (Figures 5E and 5F).

### SUMO-2 Promotes Accumulation of Insoluble Mutant HTT

Emerging data suggest that SUMOylation may influence aggregation and accumulation of aggregation-prone neurodegenerative disease proteins (Kim et al., 2011; Tatham et al., 2011). To address the functional consequences of SUMO modification of mutant HTT on disease, the involvement of SUMO-1 and SUMO-2 modification on the formation of insoluble HTT species was evaluated in HeLa cells, where SUMO modification systems are highly active. Our previous studies evaluated visible inclusion formation and levels of soluble mutant HTT and showed that fusion of SUMO to the HTTex1p N terminus promoted stabilization of HTTex1p (Steffan et al., 2004). However, we and others have since identified the HTTex1p N terminus as an important mediator of aggregation, localization, and protein stability (Atwal and Truant, 2008; Rockabrand et al., 2007; Sivanandam et al., 2011; Thompson et al., 2009; Zheng et al., 2013), which may have been masked by the presence of the SUMO moiety. SUMO-1 also decreased mutant HTT aggregation in the presence of Rhes and increased toxicity in cells (Subramaniam et al., 2009). In each case, only SUMO-1 was evaluated.

**Figure 6:**
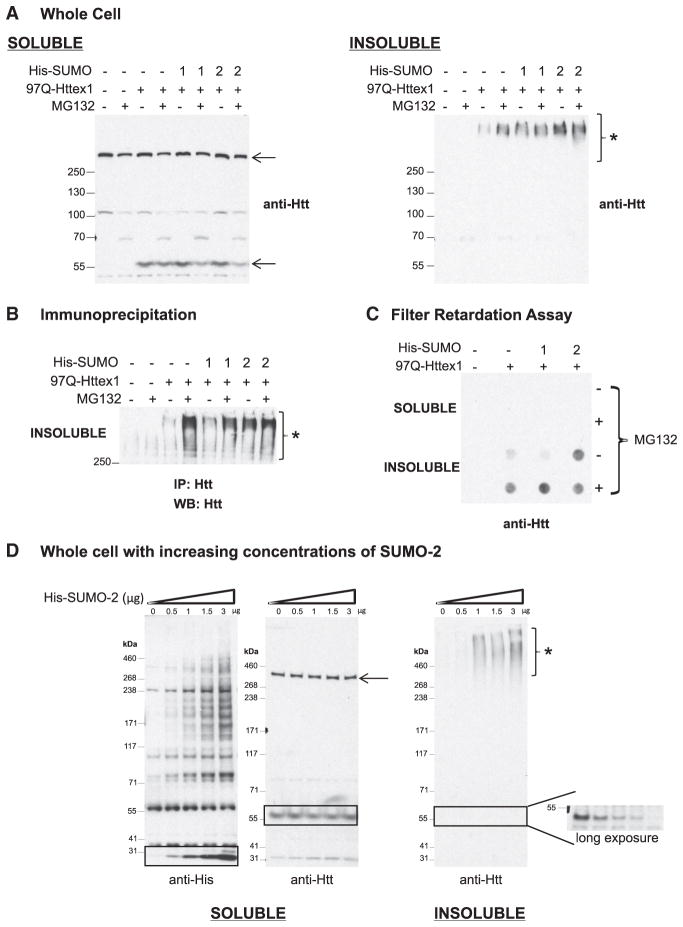
SUMO-2 Causes Mutant HTT to Accumulate (A) Western analysis of whole-cell lysates from HeLa cells transfected with His-SUMO-1 or SUMO-2 and/or 97Q-HTT exon 1 and treated with 5 μM MG132 for 18 hr. Lysates were separated using differential centrifugation into a detergent-soluble fraction (SOLUBLE) with 1% Triton X-100 and a detergent-insoluble fraction (INSOLUBLE) with 4% SDS. Western blot probed with anti-HTT shows full-length endogenous HTT in the SOLUBLE fraction (upper arrow) and 97Q-HTTex1 (lower arrow) (left panel). In the INSOLUBLE fraction, HTT HMW species are indicated by the bracket and asterisks (right panel). (B) MG132 and SUMO-2 cause mutant HTT to accumulate as HMW species (bracke and asterisk). Western blot showing IP with HTT antibody crosslinked beads from the detergent-insoluble fraction probed with the anti-HTT antibody. (C) Mutant HTT (97Q-Httex1) fibrils are detected with anti-HTT in the insoluble fraction with treatment of MG132 or addition of exogenous SUMO-2. (D) Western blot with increasing concentrations of SUMO-2 detected with anti-His antibody (left panel). Middle panel is the same western blot probed with anti-HTT showing soluble forms of HTT. Right panel presents western blot from detergent-insoluble fraction with monomeric HTT (97Q) at 55 kDa, and the asterisk (*) indicates the HMW species. Note that all experiments were performed in triplicate; representative figures are shown.

For HD, the process of aggregation and specific aggregation intermediates are likely to be critical to pathogenesis. Using a centrifugation protocol published for α-synuclein (Kim et al., 2011), lysates were separated into detergent-soluble and detergent-insoluble fractions. The detergent-soluble fraction contains monomeric HTT, which includes overexpressed mutant HTTex1p (97Q) and endogenous full-length HTT (indicated by arrows in Figure 6A, SOLUBLE fraction). In contrast, the detergent-insoluble fraction contains only high molecular weight (HMW) HTT in the samples containing 97Q-HTTex1p (Figure 6A, INSOLUBLE fraction), which are likely multimers or potentially oligomers of soluble HTT.

MG132 is a proteasomal inhibitor that causes the accumulation of mutant HTT in cells (Lee and Goldberg, 1998). To investigate the relationship between proteasomal degradation and SUMO modification and to analyze levels of soluble and insoluble HTT species in the presence of SUMO-1 and SUMO-2, HeLa cells were transiently transfected with 97Q-HTTex1p and treated with MG132 (5 μM). Treatment with MG132 causes a robust increase in HMW mutant HTT in the insoluble fraction (Figure 6A), with accumulation of ubiquitin-modified cellular proteins (data not shown). In contrast, soluble, monomeric HTT levels are maintained or slightly decreased (Figure 6A), supporting the concept that impairment of proteasomal function increases levels of aggregating HTT. Immunoprecipitation was performed to increase HTT detection. Addition of SUMO-1 had little to no additional effect on soluble HTT or insoluble HMW HTT levels (Figures 6B and 6C). However, the addition of SUMO-2 caused an increase in insoluble HTT (Figures 6B and 6C) that was comparable to proteasome inhibition. This effect is not augmented by combined SUMO-2 expression and proteasome inhibition, suggesting that SUMO-2 modulates accumulation and aggregation of mutant HTT in a manner that mimics proteasome inhibition. This regulation of insoluble HTT levels by SUMO-2 is dose dependent. When cells were treated with increasing amounts of SUMO-2, verified by increasing levels of mono-SUMO-2 by western analysis (Figure 6D, boxed in first panel), increasing levels of HMW HTT were observed, whereas monomeric levels showed a corresponding decrease in the insoluble fraction, potentially reflecting insoluble monomeric HTT levels.

**Figure 7:**
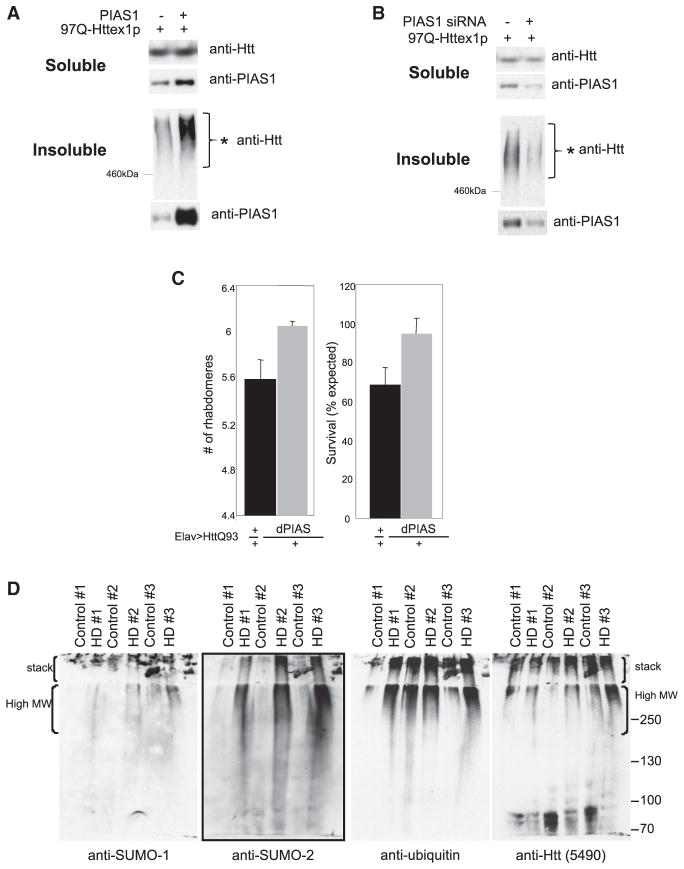
SUMO-2 Proteins Accumulate in HD Brain (A) Western blot analysis of HeLa cells over-expressing exogenous PIAS1 in the presence of mutant HTT (97Q) when separated into detergent-soluble and detergent-insoluble fractions. No difference is detected in monomeric HTT (top panel, Soluble), but HMW HTT levels increase with PIAS1 overexpression. Anti-PIAS1 antibody (Invitrogen) was used to detect PIAS1. (B) Acute knockdown of PIAS1 decreases HMW HTT species in the detergent-insoluble fraction. PIAS1 knockdown is detected in detergent-soluble and -insoluble fractions using anti-PIAS1 antibody. (C) *Drosophila melanogaster* expressing mutant HTTex1p (93Q) in a reduced Su(var)2-10/dPIAS genetic background exhibits statistically significantly reduced photoreceptor neuron degeneration (left panel, p = 0.033) when comparing *dPIAS*/+ to +/+ flies and increased overall survival (right panel, p = 0.047) when comparing *dPIAS*1/+ to +/+ flies. Significance was measured by Student’s t test. (D) HMW SUMO-2 accumulates in postmortem HD striata. Western blot analysis of the insoluble fraction from three control and three HD postmortem striata as described (Experimental Procedures).

Because SUMO-2 modulates HMW HTT species and SUMO-2 modification of HTT appears to require the presence of a SUMO E3 ligase, we tested whether PIAS1 alone can modulate mutant HTT accumulation. When PIAS1 was over-expressed in the presence of expanded repeat HTT with 97Qs, the detergent-insoluble HTT HMW “oligomeric” species increased, whereas soluble HTT appeared to be unaffected (Figure 7A), and a reduction of the HMW oligomeric HTT species is observed following PIAS1 acute knockdown (Figure 7B). Taken together, these data demonstrate that PIAS1 can regulate the accumulation of insoluble HMW HTT polypeptide species, suggesting that modulation of PIAS1 may influence pathogenesis in HD.

### Genetic Reduction of Su(-var)2-10 Is Neuroprotective in Mutant HTTex1p-Expressing *Drosophila*

To validate the potential involvement of PIAS proteins in HD pathogenesis in vivo, the single *Drosophila* PIAS protein, Su(-var)2-10 (*dPIAS*) (Hari et al., 2001), was evaluated for its effect on HD-like phenotypes in a fly model (Steffan et al., 2001). When expressed in all neurons from embryogenesis on, expanded repeat HTTex1p (93Q) causes a progressive loss of visible rhabdomeres (photoreceptor neurons in the eye) (Marsh and Thompson, 2006) and a decrease in the number of flies that eclose from the pupal case as adult flies (Figure 7C). When expressed in a background of heterozygous genetic reduction of *dPIAS*, the number of visible photoreceptor neurons and survival (eclosion) are both increased (Figure 7C). The observed neuroprotection is not simply a consequence of decreased transgene expression based on analysis of HTT RNA by qPCR (data not shown). These results are consistent with our previous observations showing that reduction of *Drosophila* SUMO (*smt3*) was protective in this same model (Steffan et al., 2004).

### Insoluble SUMO-2-Modified Proteins Are Increased in Human HD Brain

To investigate whether SUMO-modified proteins accumulate in HD brain tissue compared to control subjects and whether SUMO-1 or SUMO-2 has selective effects, postmortem striata from three control and three HD brains were evaluated. Each of the HD subjects displayed a remarkable accumulation of SUMO-2-modified protein compared to controls in insoluble fractions (Figure 7D). To a lesser extent, accumulation of SUMO-1 is also observed. Differences in the levels of SUMO-2-modified protein do not correlate with ubiquitin reactivity in control and HD brain fractions but, rather, appear to be specific for SUMO. Although we cannot conclude from these data that the increased SUMO-2 reactivity represents an increase in mutant HTT SUMO-2 modification per se, a HTT antibody raised against aa 115–129 shows a similar pattern of increased HMW HTT species in HD samples compared to controls (Figure 7D), suggesting that HTT is included in the proteins that accumulate in HD striata. Taken together, these results support SUMO-2 relevance in HD pathology and that SUMO-modifying enzymes may be valid therapeutic targets.

## DISCUSSION

SUMO modification contributes to an impressive array of regulatory mechanisms that have critical biological functions (Bruderer et al., 2011). In turn, dysregulation of this cellular process is implicated in diseases ranging from cancer to neurological disease (Gareau and Lima, 2010). Although SUMO modification is transient, downstream consequences are long lasting and impact processes such as protein folding, subcellular localization, stability, transcriptional regulation, and protein activity (Geiss-Friedlander and Melchior, 2007), all of which are affected in HD (Zheng and Diamond, 2012). Implicating a role in neurodegenerative diseases, a growing number of causative neurodegenerative disease proteins either colocalize with SUMO molecules or are target proteins for SUMO modification (for review, see Krumova and Weishaupt, 2013; Wilkinson et al., 2010). To date, the primary mechanisms involve altered solubility of or visible inclusion formation by these disease proteins, with ensuing protective (SBMA), deleterious (SCA7), or mixed effects (α-synuclein), depending on the protein context and form of SUMO tested. Enzymes involved in these processes, such as Rhes, are beginning to emerge (Oh et al., 2011; Subramaniam et al., 2009).

Here, we demonstrate that SUMO-2 modification of HTT, a stress-responsive modification pathway not previously investigated for HTT, regulates the accumulation of insoluble mutant HTT. This SUMO form is consistently upregulated in striata from several HD mouse models, at a stage of disease anticipated to display significant dysregulation of protein homeostasis network components. Furthermore, PIAS1, which selectively enhances HTT SUMO-1 and SUMO-2 modification and is expressed in brain, is integral to this accumulation. The functional relevance of these findings is further validated by (1) the neuroprotection observed upon reduction of the single *Drosophila* PIAS (dPIAS), which is most similar to PIAS1 (Hari et al., 2001), in flies expressing a mutant fragment of HTT, and (2) the profound accumulation of SUMO-2-modified HMW protein in human HD brain. The finding that the stress-responsive SUMO-2 is likely most relevant to HD pathogenesis over basal SUMO-1 modification by regulating the accumulation of HMW and likely poly-SUMOylated protein is consistent with the prevailing literature that chronic expression of mutant HTT causes cellular stress, including oxidative stress (Turner and Schapira, 2010). This cellular stress, which is likely progressive and could therefore promote stress responses, including SUMO-2 modification, could then contribute to disease. Validation of SUMO-2 involvement in response to neuronal stressors has recently emerged in several systems, including APP overexpression in AD mice (McMillan et al., 2011), transient cerebral ischemia in brains of ground squirrels (Lee et al., 2012), and transient ischemia in cells (Yang et al., 2012), in some cases, promoting a neuroprotective response to ischemic stress (Datwyler et al., 2011).

We previously reported that mimicking phosphorylation of S13 and S16 reduces monoSUMOylation and increases polySUMOylation of mHTTex1p with a highly expanded 97Q repeat (Thompson et al., 2009). Here, we confirm that K6 and K9 within the first 17 aa domain are indeed SUMO-1 modification sites even though these lysines do not lie within classic SUMO consensus sequences. Intriguingly, SUMO-1 is conjugated to K6 and K9 equivalently in the absence of other modifications, whereas phosphomimetic substitutions of S13 and S16 appear to block SUMO modification on K9 or promote SUMO-1 modification on K6, which may be significant to regulation of other HTT modifications, such as K9 acetylation. In cells, mimicking phosphorylation at these sites enhances SUMO-2 modification in the presence of a relevant E3 SUMO ligase. Given that phosphorylation of HTT is responsive to inflammatory cues (Thompson et al., 2009), that PIAS1 is involved in immune function, and that inflammation is increased in HD (Björkqvist et al., 2009; Khoshnan et al., 2004), it is likely that SUMO-2 modification is also responsive to inflammatory cues that appear early in HD.

A key feature in HD is the accumulation of mutant HTT fragments containing the polyQ expansion (Landles et al., 2010). WT and mutant HTT can be cleaved by caspases, calpains, and aspartyl proteases to form N-terminal fragments (Warby et al., 2008), which become toxic when in the context of the expanded polyglutamine repeat. Studies in mice expressing a caspase-6-resistant form of mutant HTT suggest that HTT proteolysis specifically at 586 may be critical to HD pathogenesis (Graham et al., 2006; Warby et al., 2009), and overexpression of transgenic-expanded repeat HTT 586 supports potential toxicity of this fragment (Waldron-Roby et al., 2012). Analysis of longer polyQ polypeptides revealed that unexpanded and expanded HTT 586 fragments are SUMOylated downstream of HTTex1p. The caspase-6 cleavage fragment of HTT (586 aa) has five predicted SUMOylation sites C-terminal to exon 1, with potentially greater than 13 overlapping SIMs within this region, depending on the SIM evaluated. SIMs are noncovalent protein-protein interactions that have recently emerged as having critical regulatory properties (Gareau and Lima, 2010). For example, the ubiquitin ligase RNF4 has multiple SIMs that recognize polySUMO-2 chains and ubiquitinate them for degradation by the proteasome (Geoffroy and Hay, 2009; Tatham et al., 2008). Indeed, these SIMs may be important signal transduction inducers downstream of polySUMOylation events (Sun and Hunter, 2012). SIMs within target proteins can also enhance their SUMO modification and are found within several E3-SUMO ligases, including PIAS1 (Gareau and Lima, 2010). The implications of these multiple SIMs in HTT are not yet clear; however, we are actively pursuing this area of investigation.

The interplay between SUMO-2 and proteasome inhibition is consistent with recent proteomic analysis of extracts from HeLa cells treated with MG132 to identify SUMO-2-modified proteins (Tatham et al., 2011). In these studies, all SUMO paralogs accumulated upon treatment with MG132; however the greatest response exhibited was by SUMO-2, suggesting that SUMO modification of cellular proteins is not only involved in regulating proteostasis of unfolded and misfolded proteins within a cell but may in fact represent a response to the presence of misfolded or oligomerized proteins, such as mutant HTT, and be involved in protein clearance mechanisms. This hypothesis is supported by studies showing that the presence of mutant HTT polypeptide alone in *C. elegans* can cause the misfolding and inactivation of temperature-sensitive mutant proteins to a similar degree as heat shock (Gidalevitz et al., 2006). These findings suggested that mutant HTT protein expression is sufficient to impact the protein homeostatic network and relevant to the work described here, accumulation of SUMO-2-modified cellular proteins. Furthermore, when the production of misfolded proteins exceeds the capacity of the chaperone and UPS systems, mimicked here by proteasome/cathepsin inhibition by MG132, then these proteins may be targeted for degradation by autophagy, which also becomes impaired late in disease. As protein clearance mechanisms become impaired upon aging, modified proteins normally targeted for degradation by post-translational modification, such as phosphorylation and acetylation, may accumulate and take on toxic functions. Supporting this concept, proteasome inhibition promoted formation of aggregates containing SUMO-modified α-synuclein (Kim et al., 2011).

In summary, the work presented here supports a general mechanism in HD whereby the chronic expression of expanded repeat HTT promotes general protein misfolding and initiation of stress response pathways that promote SUMO-2 modification of HTT, progressively resulting in accumulation of insoluble and HMW species that may be a reflection of ongoing pathogenesis. In addition, loss of normal HTT functions may also contribute to the accumulation of SUMOylated proteins (Steffan, 2010). Initially, SUMO-2 modification and polySUMOylation are likely to facilitate normal cellular clearance mechanisms with integration between SUMOylation and ubiquitination and serve in a neuroprotective capacity; however, these are likely to become toxic as pathways become impaired and these species accumulate and cause further disruption of overall cellular protein homeostasis, reflected here by increased expression and level of SUMO-2 and other SUMO modification cellular components. This is demonstrated by the accumulation of SUMO-2 protein in a HMW insoluble fraction from human HD striatum, the region most profoundly affected in HD. We further identify a HTT E3-SUMO ligase, PIAS1, which is expressed in relevant brain regions and appears to have a pivotal role in the regulation of SUMO-2-modified HTT, providing a novel and selective therapeutic target.

## EXPERIMENTAL PROCEDURES

### Plasmids

Plasmid generation is described in the Supplemental Information.

### siRNA

siRNA against PIAS1 and a nontargeting control were purchased from Dharmacon (Thermo Scientific) and include PIAS1-ON-TARGETplus SMARTpool (L-008167-00-0005), Human PIAS1, NM_016166, and ON-TARGETplus Non-targeting siRNA #1 D-001810-01-20.

### Primary Antibodies

Anti-HTT (Enzo Life Sciences) antibody was generated in collaboration with England Enzo Life Sciences UK (Palatine House, Matford Court, Exeter). The following antibodies were also used: anti-HTT (MAB5490) and anti-HA.11 Clone 16B12 monoclonal (Covance); anti-Myc 9E10 (Millipore); anti-EGFP detected by Living Colors Full-Length Monoclonal Antibody (Clontech; JL-8); anti-mPIAS1 (Invitrogen); anti-SUMO-1 (Enzo Life Sciences); anti-SUMO-2 (MBL International); and anti-ubiquitin (Santa Cruz Biotechnology).

### GST Pull-Down Assays

Assays were performed as previously described (Steffan et al., 2000).

### In Vitro SUMO Modification

The expression and purification of human E1 (SAE1/UBA2ΔCT), E2 (UBC9), IR1* (RanBP2, aa 2,631–2,695), and mature SUMO-1 have been previously described (Bernier-Villamor et al., 2002; Lois and Lima, 2005; Olsen et al., 2010; Reverter and Lima, 2005).

### Mass Spectrometry

SUMO-1 T95R was generated by PCR mutagenesis (QuikChange Site-Directed Mutagenesis Kit; Stratagene) to introduce a trypsin cleavage site near the SUMO C terminus and purified as described for SUMO-1. Reactions containing 200 μM SUMO-1 T95R, 200 μM HTT (WT or S13,16D), 200 nM E1, 200 nM E2, and 2 μM IR1* in reaction buffer (20 mM HEPES [pH 7.5], 50 mM NaCl, 5 mM MgCl–2, 1 mM DTT, 1 mM ATP, and 0.01% Tween 20) were incubated at 37ºC, and aliquots were removed at 0 and 60 min. Samples were enriched by incubation with Ni-NTA beads (QIAGEN) and eluted with loading buffer containing 100 mM EDTA. Samples were resolved by SDS-PAGE and stained with Coomassie blue. Bands corresponding to HTT and SUMO-modified HTT were submitted for analysis by mass spectrometry (Extended Experimental Procedures).

### Cell Culture

HeLa cells were plated on 10 cm plates and cultured in DMEM plus 10% FBS. For cDNA only, cells were plated on day 1 and transiently transfected with 6 μg of total DNA and 8 μl Lipofectamine 2000 (Invitrogen) on day 2, media were changed on day 3, and cells were collected on day 4. For siRNA only, transient transfections were carried out as described above, except that 720 pmol siRNA and 36 μl of RNAi Max were used. For combined siRNA and cDNA experiments, cells were transfected with siRNA, media were replaced on day 2, and cDNA was transfected 12 hr after siRNA. Days 3 and 4 are as described above. Cells were transfected at ~70% confluency for DNA and ~30%–50% confluency for siRNA.

### Western Blot Analysis

A total of 8% bis-acrylamide gels and Invitrogen 4%–12% bis-tris mini gels were used for SDS-PAGE, proteins were transferred to nitrocellulose membrane, and nonspecific proteins were blocked with SuperBlock Blocking Buffer (Thermo Scientific). Two types of detection were used: chemiluminescence/film, or Odyssey Imager/LI-COR (Extended Experimental Procedures). Experiments were performed in triplicate with representative images shown.

### Nickel Purifications

His-tagged HTT proteins were purified using magnetic Ni-NTA nickel beads (QIAGEN). For in vitro SUMOylation assays, ST14.A cells were transfected with the 25QP-HBH constructs into ~25 10 cm plates per construct and purified under native conditions using the recommended buffers from QIAGEN (lysis buffer: 50 mM NaH_2_PO_4_, 300 mM NaCl, 10 mM imidazole, 0.05% Tween 20 [pH 8]; wash buffer: 50 mM NaH_2_PO_4_, 300 mM NaCl, 20 mM imidazole, 0.05% Tween 20 [pH 8]). For denaturing SUMO cell culture assay, HeLa cells were transfected and purified under denaturing conditions using the recommended buffer from QIAGEN (lysis buffer: 6 M guanidine HCl, 0.1 M NaH_2_PO_4_, 10 mM Tris-Cl [pH 8]; Wash Buffer #1: 8 M urea, 100 mM NaH_2_PO_4_, 10 mM Tris-Cl [pH 8]; and Wash buffer #2: 8 M urea, 100 mM NaH_2_PO_4_, 10 mM Tris-Cl [pH 6.3]). Of each lysate, 10% was subjected to TCA precipitation. For the nickel purification, 50 μl of magnetic bead slurry was added to each sample and incubated at room temperature for 3 hr. Beads were collected on the magnetic rack, washed two times with Wash Buffer #1 (8 M urea [pH 8]), one time with Wash Buffer #2 (8 M urea [pH 6.3]), and one final time with 1× PBS. Washed beads were submerged in 2× Laemmli sample buffer, boiled for 10 min, and analyzed using western analysis. Each experiment was performed in triplicate, and a representative figure is shown.

### Immunoprecipitations

HeLa cells were lysed in buffer containing 50 mM Tris-HCl [pH 8], 150 mM NaCl, 1% NP40 alternative, a mini protease inhibitor pellet (Roche), 2 mM DTT, and 25 mM N-ethylmaleimide (NEM) (Ulrich, 2009). HTT protein was either precipitated using protein G plus from Santa Cruz or hydrazide-link beads from Bioclone.

### Soluble/Insoluble Fractionation

HeLa cells were collected in lysis buffer containing 10 mM Tris (pH 7.4), 1% Triton X-100, 150 mM NaCl, 10% glycerol, and 0.2 mM PMSF (Roche Complete Protease Mini and PhosphoStop pellets). Cells collected were lysed on ice for 60 min before centrifugation at 15,000 × *g* for 20 min at 4ºC. Supernatant was collected as the detergent-soluble fraction. The pellet was washed 3× with lysis buffer and centrifuged at 15,000 × *g* for 5 min each at 4ºC. The pellet was resuspended in lysis buffer supplemented with 4% SDS, sonicated 3×, boiled for 30 min, and collected as the detergent-insoluble fraction. Protein concentration was quantitated using Lowry Protein Assay (Bio-Rad) Soluble/insoluble fractionation protocol was previously described (Kim et al., 2011).

### Filter Retardation Assay

A total of 30 μg of detergent-soluble and detergent-insoluble protein in 200 μl of 2% SDS was boiled for 5 min and run through a dot blot apparatus under a vacuum onto a cellulose acetate membrane. Membrane was then washed 3× with 0.1% SDS and then blocked in 5% milk and subject to western blot analysis (previously described by Sontag et al., 2012; Wanker et al. 1999).

### RNA Isolation and Real-Time Quantitative PCR

Cortex and striatum were dissected from 4-, 8-, and 12-week-old female WT and R6/2 mice. At King’s College London, hemizygous R6/2 mice were bred by backcrossing R6/2 males to (CBA × C57BL/6) F1 females (B6CBAF1/ OlaHsd, Harlan Olac, Bicester) and maintained as previously described by Labbadia et al. (2011). The CAG repeat was 204.7 ± 5.8. Brain tissues were homogenized in TRIzol (Invitrogen), and total RNA was isolated using RNEasy Mini kit (QIAGEN). DNase treatment was incorporated into the RNEasy procedure in order to remove residual DNA. Reverse transcription was performed using oligo (dT) primers and 1 μg of total RNA using SuperScript III First-Strand Synthesis System (Invitrogen). Quantitative PCR (qPCR) was performed (Table S1).

### Automated Y2H Screening

GATEWAY technology (Invitrogen) was used to subclone 25Q-586 and 73Q-586 aa cDNAs encoding human HTT fragments into Y2H expression plasmids. “Gateway compatible” cDNAs encoding selected proteins were generated by PCR amplification. Amplified DNA products were isolated from agarose gels and combined with the pDONR221 plasmid (Invitrogen), creating the desired entry DNA plasmids. The identity of all PCR products was verified by DNA sequencing. Subsequently, utilizing LR recombination, pBTM116_D9 plasmids (for the production of LexA DNA-binding domain fusions) were generated encoding HTT bait proteins for automated Y2H interaction mating. The identity of the plasmids was verified by BsrGI restriction digestion. Bait plasmids were transformed into the L40ccua MATa yeast strain, and yeast clones were individually mated against a matrix of MATα yeast clones encoding 16,888 prey proteins (with Gal4 activation domain fusions) using pipetting and spotting robots. The automated Y2H screenings were repeated three times. Interaction mating experiments and imaging were performed as described previously by Stelzl et al. (2005).

### *Drosophila* Crosses

Flies were reared on standard cornmeal molasses medium at 25ºC. To compare phenotypes of HTT-expressing animals in a normal versus a Su(var)2-10 (dPIAS)-reduced background, *w/w; +/+; UAS > HTTex1p-Q93/UAS > HTTex1p-Q93* females were crossed to *elav-GAL4/Y; Su(var)2-10[zimp-2]/ Sp* males. Eclosion data from ≥1,500 segregants were calculated as a percentage of *elav*-*GAL4*/+; *Su(var)2-10*/+ *HTT*/+ or *elav*-*GAL4*/+; +/*Sp; HTT*/+ flies versus HTT-nonexpressing male siblings. Pseudopupil analysis was carried out on 7-day-old flies as described (Steffan et al., 2001).

### Human Postmortem Brain

Human autopsy brain tissue, from the striatum of control and patients with HD, was obtained from individuals with ages at autopsy from 72 to 93 years of age and grades 3–4 and flash frozen with postmortem intervals ranging from 13 to 22 hr. Frozen human brain tissue was homogenized on ice using T-PER Tissue Protein Extraction Reagent (Thermo Scientific) containing a complete mini pellet (Roche), phosphatase inhibitor #1 (Sigma-Aldrich), phosphatase inhibitor #2 (Sigma-Aldrich), and 25 mM NEM. Lysates were ultracentrifuged at 45,000 rpm at 4ºC for 60 min, and the pellet was homogenized on ice in 70% formic acid, ultracentrifuged at 45,000 rpm at 4ºC for 60 min, and the supernatant was collected as the “insoluble fraction.”

## Supplementary Material

Supplemental information
