# Supplementary material for: SUMO-2 and PIAS1 Modulate Insoluble Mutant Huntingtin Protein Accumulation
Source: Cell Rep. Author manuscript; Available in PMC 2014 Feb 21. (PMC3931302; doi:10.1016/j.celrep.2013.06.034)
Supplement: Supplemental information [file NIHMS554532-supplement-Supplemental_information.pdf]

A.

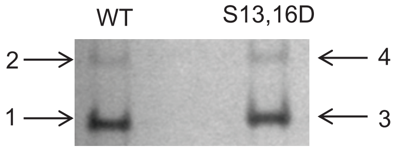

B.

1) HTT WT (25Q-HBH)

Matched peptides shown in **Bold Red**

```
  1 MATLEKLMKA FESLKSFQQQ QQQQQQQQQQ QQQQQQQQQQ QQPPPPPPPP
 51 PPPQLPQPPP QAQPLLPQPQ PPPPPPPPPP GPAVAEEPLH RPGSLINRGS
101 HHHHHHAGKA GEGEIPAPLA GTVSKILVKE GDTVKAGQTV LVLEAMKMET
151 EINAPTDGKV EKVLVKERDA VQGGQGLIKT GVHHHHHH
```

```
Start-End Observed Mr(expt) Mr(calc) Delta  Sequence
  2 - 9   496.2889 990.5632 990.5419 0.0213 M.ATLEKLMK.A
```

MS/MS Fragmentation

```
Monoisotopic mass of neutral peptide Mr(calc): 990.5419
Variable modifications:
N-term : Acetyl (Protein N-term)
M7 : Oxidation (M), with neutral losses 63.9983(shown in table), 0.0000
Ions Score: 29 Expect: 0.0011
Matches : 30/102 fragment ions using 57 most intense peaks
```

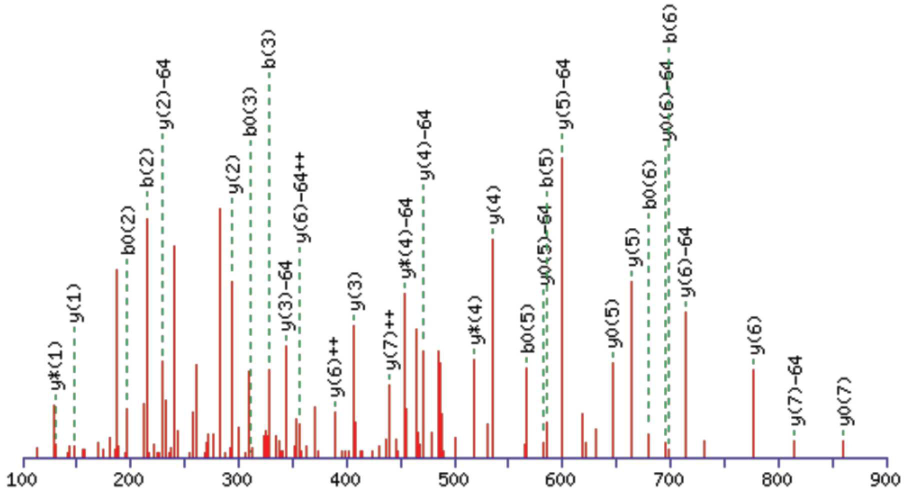

#	b	b++	b*	b*++	b0	b0++	Seq.	y	y++	y*	y*++	y0	y0++	#
1	114.0550	57.5311					A							8
2	**215.1026**	108.0550			197.0921	99.0497	T	***814.5033***	407.7553	797.4767	399.2420	796.4927	398.7500	7
3	**328.1867**	164.5970			310.1761	155.5917	L	***713.4556***	357.2314	696.4290	348.7182	695.4450	348.2262	6
4	457.2293	229.1183			439.2187	220.1130	E	***600.3715***	300.6894	583.3450	292.1761	582.3610	291.6841	5
5	**585.3243**	293.1658	568.2977	284.6525	567.3137	284.1605	K	***471.3289***	236.1681	454.3024	227.6548			4
6	**698.4083**	349.7078	681.3818	341.1945	680.3978	340.7025	L	***343.2340***	172.1206	326.2074	163.6074			3
7	781.4454	391.2264	764.4189	382.7131	763.4349	382.2211	M	***230.1499***	115.5786	213.1234	107.0653			2
8							K	***147.1128***	74.0600	130.0863	65.5468			1

Supplemental Figure 1 (continued)

B. (continued)

2) HTT WT (25Q-HBH) + SUMO T95R

Matched peptides shown in **Bold Red**

```
  1 MATLEKLMKA FESLKSFQQQ QQQQQQQQQQ QQQQQQQQQQ QQPPPPPPPP
 51 PPPQLPQPPP QAQPLLPQPQ PPPPPPPPPP GPAVAEEPLH RPGSLINRGS
101 HHHHHHAGKA GEGEIPAPLA GTVSKILVKE GDTVKAGQTV LVLEAMKMET
151 EINAPTDGKV EKVLVKERDA VQGGQGLIKT GVHHHHHH
```

```
Start-End Observed Mr(expt)  Mr(calc)   Delta  Sequence
  2 - 9   553.3118 1104.6091 1104.5849  0.0243 M.ATLEKLMK.A
```

MS/MS Fragmentation

```
Monoisotopic mass of neutral peptide Mr(calc): 1104.5849
Variable modifications:
N-term : Acetyl (Protein N-term)
K5 : GlyGly (K)
M7 : Oxidation (M), with neutral losses 0.0000(shown in table), 63.9983
Ions Score: 56 Expect: 2.3e-006
Matches : 19/102 fragment ions using 26 most intense peaks
```

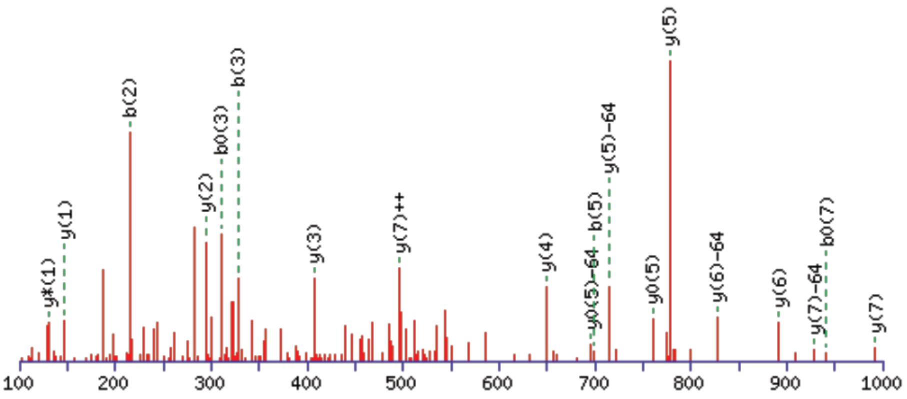

#	b	b++	b*	b*++	b0	b0++	Seq.	y	y++	y*	y*++	y0	y0++	#
1	114.0550	57.5311					A							8
2	**215.1026**	108.0550			197.0921	99.0497	T	***992.5445***	496.7759	975.5179	488.2626	974.5339	487.7706	7
3	**328.1867**	164.5970			310.1761	155.5917	L	***891.4968***	446.2520	874.4703	437.7388	873.4863	437.2468	6
4	457.2293	229.1183			439.2187	220.1130	E	***778.4128***	389.7100	761.3862	381.1967	760.4022	380.7047	5
5	**699.3672**	350.1872	682.3406	341.6740	681.3566	341.1819	K	***649.3702***	325.1887	632.3436	316.6754			4
6	812.4512	406.7293	795.4247	398.2160	794.4407	397.7240	L	***407.2323***	204.1198	390.2057	195.6065			3
7	959.4866	480.2470	942.4601	471.7337	941.4761	471.2417	M	***294.1482***	147.5777	277.1217	139.0645			2
8							K	***147.1128***	74.0600	130.0863	65.5468			1

Supplemental Figure 1 (continued)

B. (continued)

2) HTT WT (25Q-HBH) + SUMO T95R

Matched peptides shown in **Bold Red**

```
  1 MATLEKLMKA FESLKSFQQQ QQQQQQQQQQ QQQQQQQQQQ QQPPPPPPPP
 51 PPPQLPQPPP QAQPLLPQPQ PPPPPPPPPP GPAVAEEPLH RPGSLINRGS
101 HHHHHHAGKA GEGEIPAPLA GTVSKILVKE GDTVKAGQTV LVLEAMKMET
151 EINAPTDGKV EKVLVKERDA VQGGQGLIKT GVHHHHHH
```

```
Start-End Observed Mr(expt)  Mr(calc)   Delta  Sequence
  7 - 15   598.8076 1195.6007 1195.6271 -0.0264 K.LMKAFESLK.S
```

MS/MS Fragmentation

```
Monoisotopic mass of neutral peptide Mr(calc): 1195.6271
Variable modifications:
M2 : Oxidation (M), with neutral losses 63.9983(shown in table), 0.0000
K3 : GlyGly (K)
Ions Score: 12 Expect: 0.066
Matches : 21/116 fragment ions using 81 most intense peaks
```

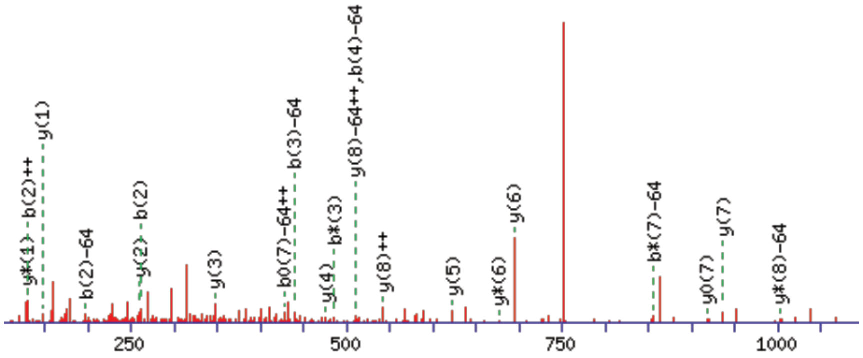

#	b	b++	b*	b*++	b0	b0++	Seq.	y	y++	y*	y*++	y0	y0++	#
1	114.0913	57.5493					L							9
2	**197.1284**	99.0679					M	1019.5520	510.2796	1002.5255	501.7664	1001.5415	501.2744	8
3	**439.2663**	220.1368	422.2398	211.6235			K	***936.5149***	468.7611	919.4884	460.2478	918.5043	459.7558	7
4	**510.3035**	255.6554	493.2769	247.1421			A	***694.3770***	347.6921	677.3505	339.1789	676.3665	338.6869	6
5	657.3719	329.1896	640.3453	320.6763			F	***623.3399***	312.1736	606.3134	303.6603	605.3293	303.1683	5
6	786.4145	393.7109	769.3879	385.1976	768.4039	384.7056	E	***476.2715***	238.6394	459.2449	230.1261	458.2609	229.6341	4
7	873.4465	437.2269	856.4199	428.7136	855.4359	428.2216	S	***347.2289***	174.1181	330.2023	165.6048	329.2183	165.1128	3
8	986.5306	493.7689	969.5040	485.2556	968.5200	484.7636	L	***260.1969***	130.6021	243.1703	122.0888			2
9							K	***147.1128***	74.0600	130.0863	65.5468			1

Supplemental Figure 1 (continued)

C.

3) HTT Phosphomimic (25Q-S13,S16-HBH)

Matched peptides shown in **Bold Red**

```
  1 MATLEKLMKA FEDLKDFQQQ QQQQQQQQQQ QQQQQQQQQQ QQPPPPPPPP
 51 PPPQLPQPPP QAQPLLPQPQ PPPPPPPPPP GPAVAEEPLH RPGSLINRGS
101 HHHHHHAGKA GEGEIPAPLA GTVSKILVKE GDTVKAGQTV LVLEAMKMET
151 EINAPTDGKV EKVLVKERDA VQGGQGLIKT GVHHHHHH
```

```
Start-End Observed Mr(expt)  Mr(calc)   Delta  Sequence
  2 - 9   496.2847   990.5548  990.5419  0.0128 M.ATLEKLMK.A
```

MS/MS Fragmentation

```
Monoisotopic mass of neutral peptide Mr(calc): 990.5419
Variable modifications:
N-term : Acetyl (Protein N-term)
M7 : Oxidation (M), with neutral losses 0.0000(shown in table), 63.9983
Ions Score: 31 Expect: 0.00071
Matches : 9/102 fragment ions using 14 most intense peaks
```

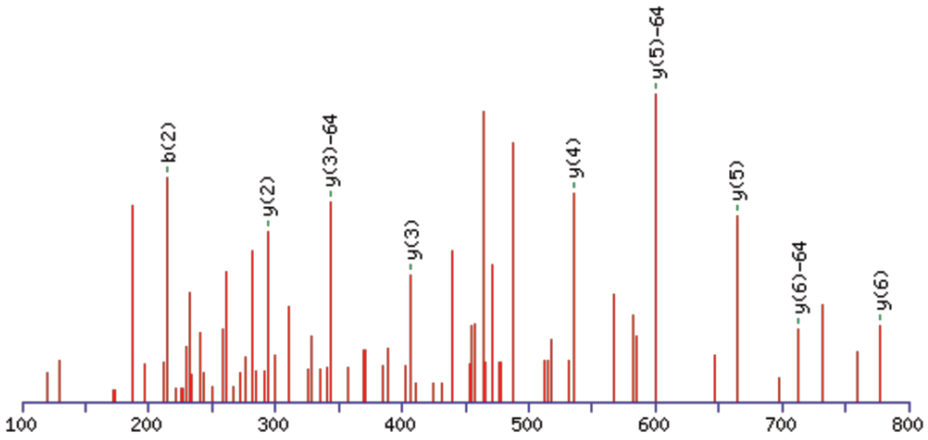

#	b	b^{++}	b^{*}	b^{*++}	b^{0}	b^{0++}	Seq.	y	y^{++}	y^{*}	y^{*++}	y^{0}	y^{0++}	#
1	114.0550	57.5311					A							8
2	215.1026	108.0550			197.0921	99.0497	T	878.5016	439.7544	861.4750	431.2411	860.4910	430.7491	7
3	328.1867	164.5970			310.1761	155.5917	L	***777.4539***	389.2306	760.4273	380.7173	759.4433	380.2253	6
4	457.2293	229.1183			439.2187	220.1130	E	***664.3698***	332.6886	647.3433	324.1753	646.3593	323.6833	5
5	585.3243	293.1658	568.2977	284.6525	567.3137	284.1605	K	***535.3272***	268.1673	518.3007	259.6540			4
6	698.4083	349.7078	681.3818	341.1945	680.3978	340.7025	L	***407.2323***	204.1198	390.2057	195.6065			3
7	845.4437	423.2255	828.4172	414.7122	827.4332	414.2202	M	***294.1482***	147.5777	277.1217	139.0645			2
8							K	147.1128	74.0600	130.0863	65.5468			1

Supplemental Figure 1 (continued)

C. (continued)

4) HTT Phosphomimic (25Q-S13,S16-HBH) + SUMO T95R

Matched peptides shown in **Bold Red**

```
  1 MATLEKLMKA FEDLKDFQQQ QQQQQQQQQQ QQQQQQQQQQ QQPPPPPPPP
 51 PPPQLPQPPP QAQPLLPQPQ PPPPPPPPPP GPAVAEEPLH RPGSLINRGS
101 HHHHHHAGKA GEGEIPAPLA GTVSKILVKE GDTVKAGQTV LVLEAMKMET
151 EINAPTDGKV EKVLVKERDA VQGGQGLIKT GVHHHHHH
```

```
Start-End Observed Mr(expt)  Mr(calc)    Delta   Sequence
  2 - 9   553.3252 1104.6358 1104.5849 0.0510  M.ATLEKLMK.A
```

MS/MS Fragmentation

```
Monoisotopic mass of neutral peptide Mr(calc): 1104.5849
Variable modifications:
N-term : Acetyl (Protein N-term)
K5 : GlyGly (K)
M7 : Oxidation (M), with neutral losses 0.0000(shown in table), 63.9983
Ions Score: 55 Expect: 3.1e-006
Matches : 21/102 fragment ions using 27 most intense peaks
```

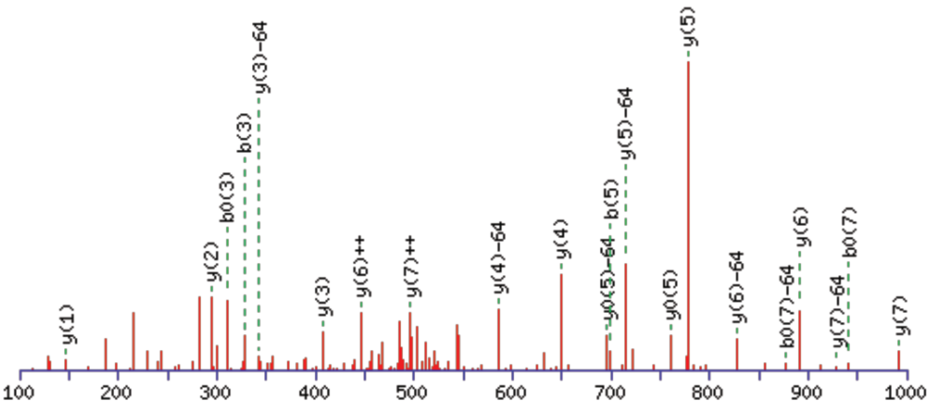

#	b	b++	b*	b*++	b0	b0++	Seq.	y	y++	y*	y*++	y0	y0++	#
1	114.0550	57.5311					A							8
2	215.1026	108.0550			197.0921	99.0497	T	***992.5445***	496.7759	975.5179	488.2626	974.5339	487.7706	7
3	**328.1867**	164.5970			310.1761	155.5917	L	***891.4968***	446.2520	874.4703	437.7388	873.4863	437.2468	6
4	457.2293	229.1183			439.2187	220.1130	E	***778.4128***	389.7100	761.3862	381.1967	760.4022	380.7047	5
5	**699.3672**	350.1872	682.3406	341.6740	681.3566	341.1819	K	***649.3702***	325.1887	632.3436	316.6754			4
6	812.4512	406.7293	795.4247	398.2160	794.4407	397.7240	L	***407.2323***	204.1198	390.2057	195.6065			3
7	959.4866	480.2470	942.4601	471.7337	941.4761	471.2417	M	***294.1482***	147.5777	277.1217	139.0645			2
8							K	***147.1128***	74.0600	130.0863	65.5468			1

Supplemental Figure 1 (continued)

C. (continued)

4) HTT Phosphomimic (25Q-S13,S16-HBH) + SUMO T95R

Matched peptides shown in **Bold Red**

1 M**ATLEKLMKA FEDLK**DFQQQ QQQQQQQQQQ QQQQQQQQQQ QQPPPPPPPP
51 PPPQLPQPPP QAQPLLPQPQ PPPPPPPPPP GPAVAEEPLH RPGSLINRGS
101 HHHHHHAGK**A GEGEIPAPLA GTVSKILVKE GDTVKAGQTV LVLEAMKMET**
151 **EINAPTDGKV EK**VLVK**ERDA VQGGQGLIK**T GVHHHHHH

Start-End	Observed	Mr(expt)	Mr(calc)	Delta	Sequence
7 - 15	661.8799	1321.7453	1321.6700	0.0753	K.**LMKAFEDLK**.D

MS/MS Fragmentation

Monoisotopic mass of neutral peptide Mr(calc): 1321.6700
Variable modifications:
K3 : GlyGly (K)
K9 : GlyGly (K)
Ions Score: 3 Expect: 0.47
Matches : 6/78 fragment ions using 22 most intense peaks

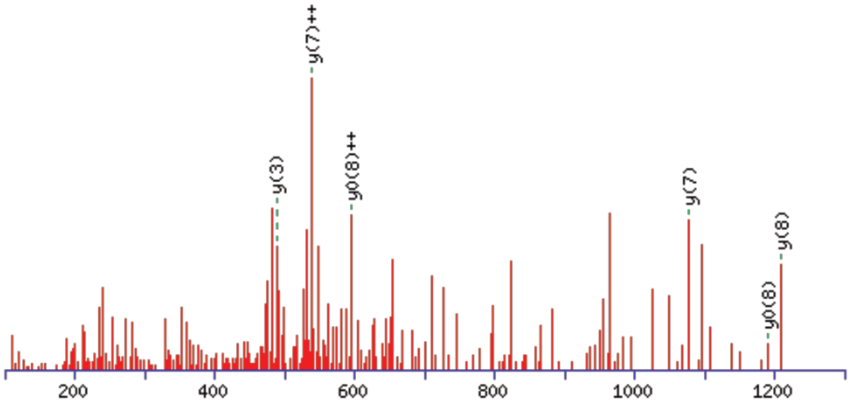

#	b	b++	b*	b*++	b0	b0++	Seq.	y	y++	y*	y*++	y0	y0++	#
1	114.0913	57.5493					L							9
2	245.1318	123.0696					M	***1209.5932***	605.3003	1192.5667	596.7870	1191.5827	596.2950	8
3	487.2697	244.1385	470.2432	235.6252			K	***1078.5527***	539.7800	1061.5262	531.2667	1060.5422	530.7747	7
4	558.3068	279.6571	541.2803	271.1438			A	836.4149	418.7111	819.3883	410.1978	818.4043	409.7058	6
5	705.3752	353.1913	688.3487	344.6780			F	765.3777	383.1925	748.3512	374.6792	747.3672	374.1872	5
6	834.4178	417.7126	817.3913	409.1993	816.4073	408.7073	E	618.3093	309.6583	601.2828	301.1450	600.2988	300.6530	4
7	949.4448	475.2260	932.4182	466.7128	931.4342	466.2207	D	***489.2667***	245.1370	472.2402	236.6237	471.2562	236.1317	3
8	1062.5288	531.7681	1045.5023	523.2548	1044.5183	522.7628	L	374.2398	187.6235	357.2132	179.1103			2
9							K	261.1557	131.0815	244.1292	122.5682			1

Supplemental Figure 1 (continued)

C. (continued)

4) HTT Phosphomimic (25Q-S13,S16-HBH) + SUMO T95R

Matched peptides shown in **Bold Red**

1 M**ATLEKLMKA FEDLK**DFQQQ QQQQQQQQQQ QQQQQQQQQQ QQPPPPPPPP
51 PPPQLPQPPP QAQPLLPQPQ PPPPPPPPPP GPAVAEEPLH RPGSLINRGS
101 HHHHHHAGKA **GEGEIPAPLA GTVSKILVKE GDTVKAGQTV LVLEAMKMET**
151 **EINAPTDGKV EK**VLVK**ERDA VQGGQGLIK**T GVHHHHHH

Start-End	Observed	Mr(expt)	Mr(calc)	Delta	Sequence
1 - 15	685.4168	2053.2284	2053.0223	0.2061	-.**MATLEKLMKAFEDLK**.D

MS/MS Fragmentation

Monoisotopic mass of neutral peptide Mr(calc): 2053.0223
Variable modifications:
N-term : Acetyl (Protein N-term)
M8 : Oxidation (M), with neutral losses 0.0000(shown in table), 63.9983
K9 : GlyGly (K)
K15 : GlyGly (K)
Ions Score: 5 Expect: 0.35
Matches : 4/234 fragment ions using 11 most intense peaks

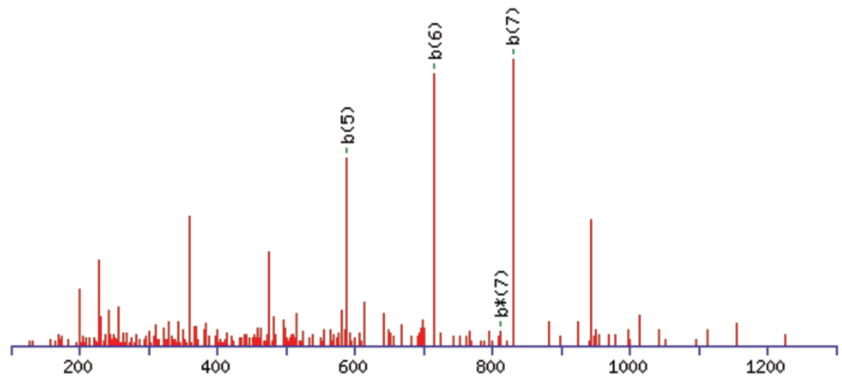

#	b	b++	b*	b*++	b0	b0++	Seq.	y	y++	y*	y*++	y0	y0++	#
1	174.0583	87.5328					M							15
2	245.0954	123.0514					A	1880.9786	940.9930	1863.9521	932.4797	1862.9681	931.9877	14
3	346.1431	173.5752			328.1326	164.5699	T	1809.9415	905.4744	1792.9150	896.9611	1791.9309	896.4691	13
4	459.2272	230.1172			441.2166	221.1119	L	1708.8938	854.9506	1691.8673	846.4373	1690.8833	845.9453	12
5	***588.2698***	294.6385			570.2592	285.6332	E	1595.8098	798.4085	1578.7832	789.8952	1577.7992	789.4032	11
6	***716.3647***	358.6860	699.3382	350.1727	698.3542	349.6807	K	1466.7672	733.8872	1449.7406	725.3740	1448.7566	724.8819	10
7	***829.4488***	415.2280	812.4223	406.7148	811.4382	406.2228	L	1338.6722	669.8397	1321.6457	661.3265	1320.6616	660.8345	9
8	976.4842	488.7457	959.4577	480.2325	958.4736	479.7405	M	1225.5881	613.2977	1208.5616	604.7844	1207.5776	604.2924	8
9	1218.6221	609.8147	1201.5955	601.3014	1200.6115	600.8094	K	1078.5527	539.7800	1061.5262	531.2667	1060.5422	530.7747	7
10	1289.6592	645.3332	1272.6327	636.8200	1271.6486	636.3280	A	836.4149	418.7111	819.3883	410.1978	818.4043	409.7058	6
11	1436.7276	718.8674	1419.7011	710.3542	1418.7171	709.8622	F	765.3777	383.1925	748.3512	374.6792	747.3672	374.1872	5
12	1565.7702	783.3887	1548.7437	774.8755	1547.7596	774.3835	E	618.3093	309.6583	601.2828	301.1450	600.2988	300.6530	4
13	1680.7972	840.9022	1663.7706	832.3889	1662.7866	831.8969	D	489.2667	245.1370	472.2402	236.6237	471.2562	236.1317	3
14	1793.8812	897.4442	1776.8547	888.9310	1775.8707	888.4390	L	374.2398	187.6235	357.2132	179.1103			2
15							K	261.1557	131.0815	244.1292	122.5682			1

Figure S1. Mass Spectrometry Analysis Identifying K6 and K9 the Primary Sites Modified by SUMO in HTTex1p

(A) Western blot of *in vitro* SUMOylation assay identifying the bands subjected to mass spectrometry. In lane 1, labeled wildtype (WT), #1 represents unmodified HTT (WT HTT) with 25Qs and a 6x His-biontinylation sequence-6x His tag (HBH) and #2 represents the SUMO-1 modified form (WT HTT with SUMO). In lane 2, labeled S13,16D, #3 represents unmodified mutant HTT ((MUT HTT-S13,16,D) with S13 and S16 mutated to Asp (D) to mimic phosphorylationand #4 identifies the S13,16D-SUMO modified form (MUT HTT-S13,16,D with SUMO).

(B) Mass spectrometry data from *in vitro* SUMOylation assay in (A). Missed cleavage along with the Gly-Gly (K) indicates SUMO modification on that Lys residue. 1) Identifies the matched peptides 25Q-HBH in **Bold Red** color. 2) 25Q-HBH with SUMO shows ion scores are high for the 2-9 peptide with a missed cleavage at K6 and the 7-15 peptide with a missed cleavage at K9.

(C) 3) Identifies the matched peptides in 25Q-S13,16D-HBH in **Bold Red** color. 4) 25Q-S13,16D-HBH with SUMO, ion scores are high for most 2-9 peptides with a missed cleavage at K6. The 1-15 peptide has a low score (5) and assumes modification at K9 and K15 as a fragment corresponding to unmodified K6 is observed (not shown). However, K15 is not modified because trypsin cleaves after K15. The 7-15 peptide is also not possible, since the mass corresponds to two SUMO modifications - K9 and K15. SUMO modification only found on K6.

*Note: no other peptides were detected with Gly-Gly (K) in the other segments of HTT constructs.

Table S1. qPCR Primers, Related to Figures 2 and S2

Sumo1-F	GTGAATCCACGTCACCATGTC
Sumo1-R	GTATCTCACTGCTATCCTGTC
Sumo2-F	GTCAATGAGGCAGATCAGATTC
Sumo2-R	CACATCAATCGTATCTTCATCC
Pias1-F	GTCTCCTACGTCACCACTAAG
Pias1-R	GCATAGGCGTCATGTGGAAG
Pias2-F	CCGTGCAGTGACTTGTACAC
Pias2-R	CTCATCCACATCAGAACAGTC
Pias3-F	GGTGAGGCAATTGACTGCAG
Pias3-R	GTAGTAGCCACTTCACTGTCG
Pias4-F	GACGCTAGTGGCCAAGATGG
Pias4-R	GTCACCAGTTCGTGCTTCAG
mActin-F	AGGTATCCTGACCCTGAAG
mActin-R	GCTCATTGTAGAAGGTGTGG
